# A host-vector toolbox for improved secretory protein overproduction in *Bacillus subtilis*

**DOI:** 10.1007/s00253-022-12062-2

**Published:** 2022-07-08

**Authors:** Anna Krüger, Norma Welsch, Alexandra Dürwald, Henrike Brundiek, Rainer Wardenga, Henning Piascheck, Hendrik G. Mengers, Jana Krabbe, Sandra Beyer, Johannes F. Kabisch, Lutz Popper, Tanno Hübel, Garabed Antranikian, Thomas Schweder

**Affiliations:** 1 Institute of Technical Microbiology, Hamburg University of Technology, Kasernenstr. 12, 21073 Hamburg, Germany; 2 Pharmaceutical Biotechnology, Institute of Pharmacy, University of Greifswald, Felix-Hausdorff-Str. 3, 17487 Greifswald, Germany; 3 Institute of Marine Biotechnology, Walther-Rathenau-Str. 49, 17489 Greifswald, Germany; 4 Enzymicals AG, Walther-Rathenau-Straße 49a, 17489 Greifswald, Germany; 5 Institute of Applied Microbiology - iAMB, Aachen Biology and Biotechnology - ABBt, RWTH Aachen University, Worringerweg 1, 52074 Aachen, Germany; 6 Department of Biomolecular Chemistry, Leibniz Institute for Natural Product Research and Infection Biology, HKI, Beutenbergstr. 11a, 07745 Jena, Germany; 7 Bioprocess Center, Eppendorf AG, Rudolf-Schulten-Str. 5, 52428 Jülich, Germany; 8 Department of Biotechnology and Food Science, NTNU, Sem Sælands vei 6, 7034 Trondheim, Norway; 9 Stern Enzym GmbH & Co. KG, Kurt-Fischer-Str. 55, 22926 Ahrensburg, Germany; 10 Miltenyi Biotec GmbH, Robert-Koch-Str. 1, 17166 Teterow, Germany

**Keywords:** *Bacillus subtilis*, Yeast sulfhydryl oxidase, Human interleukin-1β, Protease deficiency, Expression system, Fed batch, Protein secretion, Overproduction

## Abstract

**Abstract:**

Target proteins in biotechnological applications are highly diverse. Therefore, versatile flexible expression systems for their functional overproduction are required. In order to find the right heterologous gene expression strategy, suitable host-vector systems, which combine different genetic circuits, are useful. In this study, we designed a novel *Bacillus subtilis* expression toolbox, which allows the overproduction and secretion of potentially toxic enzymes. This toolbox comprises a set of 60 expression vectors, which combine two promoter variants, four strong secretion signals, a translation-enhancing downstream box, and three plasmid backbones. This *B. subtilis* toolbox is based on a tailor-made, clean deletion mutant strain, which is protease and sporulation deficient and exhibits reduced autolysis and secondary metabolism. The appropriateness of this alternative expression platform was tested for the overproduction of two difficult-to-produce eukaryotic model proteins. These included the sulfhydryl oxidase Sox from *Saccharomyces cerevisiae*, which forms reactive hydrogen peroxide and undesired cross-linking of functional proteins, and the human interleukin-1β, a pro-inflammatory cytokine. For the best performing Sox and interleukin, overproducing and secreting variants of these new *B. subtilis* toolbox fermentation strategies were developed and tested. This study demonstrates the suitability of the prokaryotic *B. subtilis* host-vector system for the extracellular production of two eukaryotic proteins with biotechnological relevance.

**Key points:**

*• Construction of a versatile Bacillus subtilis gene expression toolbox.*

*• Verification of the toolbox by the secretory overproduction of two difficult-to-express proteins.*

*• Fermentation strategy for an acetoin-controlled overproduction of heterologous proteins.*

**Supplementary Information:**

The online version contains supplementary material available at 10.1007/s00253-022-12062-2.

## Introduction

Engineered *Bacillus* strains, such as *Bacillus subtilis*, are important workhorses for the controlled overproduction of target proteins. *Bacillus subtilis* exhibits two major advantages over the widely used *Escherichia coli* expression systems. Firstly, it is a GRAS (generally recognized as safe) organism and as an endotoxin-free platform highly suitable for applications especially in the food and feed industry (Yang et al. 2016; Taguchi et al. 2015) or as vaccine biofactory (Rosales-Mendoza et al. 2016). Secondly, it enables extracellular protein production (Schallmey et al. 2004; Harwood and Cranenburgh 2008) and is considered to be a supersecreting cell factory (van Dijl and Hecker 2013). However, a drawback of available *B. subtilis* expression strains, such as *B. subtilis* WB600 or *B. subtilis* WB800 (Wu et al. 1991, 2002), is the fact that they contain several antibiotic resistance genes, which are not desired in many applications (Emond et al. 2001). Hence, aided by techniques for genomic modifications, such as the Cre-loxP technique (Kumpfmüller et al. 2013), CRISPR-Cas9 (Zhang et al. 2018), or counter-selection systems (Liu et al. 2008; Yu et al. 2010), marker-free expression strains can be generated that are favorable for various applications. Recently, new markerless *B. subtilis* expression strains were developed, which exhibited improved metabolic or protein production activities (Liu et al. 2018a, 2018b; Fan et al. 2018). Such optimized *B. subtilis* expression hosts comprise novel, tailor-made strains with reduced extracellular proteolytic activities (Zhang et al. 2018; Zhao et al. 2019). Furthermore, genome-reduced strains, like *B. subtilis* PG10, have been shown to be improved cell factories, which enable a more efficient production of “difficult-to-produce proteins” (Aguilar Suárez et al. 2019) or lantibiotics (van Tilburg et al. 2020).

Each target protein is unique and demands special expression systems or conditions. Many heterologous proteins, especially those that originate from eukaryotic organisms, are frequently difficult-to-express. Furthermore, target proteins could be harmful to the host and can therefore not be overproduced in established expression hosts. For example, sulfhydryl oxidases (Sox) are promising enzymes for food applications to improve rheological properties of doughs for pasta and pastries (Faccio et al. 2011). However, Sox is difficult-to-produce with classical microbial expression systems due to its toxicity caused by hydrogen peroxide formation. Furthermore, Sox is responsible for undesired cross-linking of functional proteins. One means to overcome this problem is to use expression systems which are specifically designed for the production of such toxic enzymes (Zemella et al. 2015).

To date, a permanently increasing number of novel *Bacillus* host-vector systems is available. One recent example for *B. subtilis* is the LIKE system (Toymentseva et al. 2012), which is based on a strictly controlled promoter that is strongly induced by cell wall-specific antibiotics. Another example is the BioBrick toolbox, which comprises essential genetic building blocks like integrative and replicative expression vectors, different promoters, or epitope tags, and thus enables a standardized protein overproduction with *B. subtilis* (Radeck et al. 2013; Popp et al. 2017). However, none of the existing systems combines satisfyingly all required parameters for an efficient expression platform, which should include (i) a suitable vector backbone, (ii) a selection marker, (iii) a strictly controlled promotor, (iv) a strong terminator, and (v) a suitable host chassis. Furthermore, strong secretion signals and translation enhancer sequences might be beneficial. Due to this variety of parameters, a simple handling and the possibility to easily exchange specific genetic elements should be a prerequisite in the development of a suitable and well-structured expression platform.

In this study, we constructed an alternative expression platform for *B. subtilis* and designed a novel host-vector system for recombinant protein production, which tries to meet the abovementioned parameters and enables the overproduction of potential toxic target proteins. The new markerless *B. subtilis* expression strain is deficient in its eight main extracellular proteases and shows reduced lysis rates by deletion of the *lytC gene* (Kabisch et al. 2013) and no sporulation by a *spoIIGA* mutation. In addition, this prototrophic *B. subtilis* strain was further adapted by deletions in three major secondary metabolite gene clusters in order to prevent interfering background metabolic activities and to support the protein downstream processing. We demonstrate that this new *B. subtilis* strain with the toolbox of 60 different expression vectors enables the development of successful strategies for the secretory overproduction of sophisticated eukaryotic proteins like the yeast sulfhydryl oxidase Sox and the human growth factor interleukin-1β.

## Materials and methods

Unless stated otherwise all chemicals used in this study were of analytical grade purchased from Carl Roth (Karlsruhe, Germany), Sigma-Aldrich (Steinheim, Germany), Fisher Scientific (Schwerte, Germany), Merck (Darmstadt, Germany), and OlbrichtArom (Leisnig, Germany). A derivative of the *B. subtilis* strain ATCC 6051 (American Type Culture Collection) was used for all chromosomal gene knockouts (Kabisch et al. 2013). *Escherichia coli* DH10B (Invitrogen, Darmstadt, Germany) [F-*end*A1 *rec*A1 *gal*E15 *gal*K16 *nup*G *rps*L Δ*lac*X74 Φ80*lac*ZΔM15 *ara*D139 Δ(*ara*,*leu*)7697 *mcr*A Δ(*mrr*-*hsd*RMS-*mcr*BC) λ-] was used as host strain for all cloning procedures. Sequences of primers used in cloning experiments of this study are summarized in Table S1. All strains created in this study are listed in Table 1. Plasmids constructed in this study are summarized in Table 2 and for the toolbox in Table S2 and S3.

**Table 1:** Overview of all *B. subtilis* strains constructed and used in this study

Strain	Relevant genotype/expression vector	Reference	Deletion
*Bs*JK32	Δ*sacA*:: (ZeoR, P_*xyl*_-*cre*, *xylR*, P_*spac*_-*comS*, *lacI*), (ATCC 6051-derivative)	This study	***sacA***
*Bs*JK49	Δ*sacA*:: (ZeoR, P_*xyl*_-*cre*, *xylR*, P_*spac*_-*comS*, *lacI*), ΔRM::lox72 (ATCC 6051-derivative)	This study	***RMS***
*Bs*JK49 Δ*lytC*	*Bs*JK49 with Δ*lytC*::lox72	This study	***lytC***
*Bs*JK49 Δ*lytC*, ΔPI	*Bs*JK49 with Δ*lytC*::lox72, Δ*bpr-spo*::lox72	This study	***bpr-spo***
*Bs*JK49 Δ*lytC*, ΔPII	*Bs*JK49 with Δ*lytC*::lox72, Δ*bpr-spo*::lox72, Δ*nprB*::lox72	This study	***nprB***
*Bs*JK49 Δ*lytC*, ΔPIII	*Bs*JK49 with Δ*lytC*::lox72, Δ*bpr-spo*::lox72, Δ*nprB*::lox72, Δ*mpr*::lox72	This study	***mpr***
*Bs*JK49 Δ*lytC*, ΔPIV	*Bs*JK49 with Δ*lytC*::lox72, Δ*bpr-spo*::lox72, Δ*nprB*::lox72, Δ*mpr*::lox72, Δ*aprE*::lox72	This study	***aprE***
*Bs*JK49 Δ*lytC*, ΔPV	*Bs*JK49 with Δ*lytC*::lox72, Δ*bpr-spo*::lox72, Δ*nprB*::lox72, Δ*mpr*::lox72, Δ*aprE*::lox72, Δ*nprE*::lox72	This study	***nprE***
*Bs*JK49 Δ*lytC*, ΔPVI	*Bs*JK49 with Δ*lytC*::lox72, Δ*bpr-spo*::lox72, Δ*nprB*::lox72, Δ*mpr*::lox72, Δ*aprE*::lox72, Δ*nprE*::lox72, Δ*vpr*::lox72	This study	***vpr***
*Bs*JK49 Δ*lytC*, ΔPVII	*Bs*JK49 with Δ*lytC*::lox72, Δ*bpr-spo*::lox72, Δ*nprB*::lox72, Δ*mpr*::lox72, Δ*aprE*::lox72, Δ*nprE*::lox72, Δ*vpr*::lox72, Δ*epr*::lox72	This study	***epr***
*Bs*JK49 Δ*lytC*, ΔPVIII	*Bs*JK49 with Δ*lytC*::lox72, Δ*bpr-spo*::lox72, Δ*nprB*::lox72, Δ*mpr*::lox72, Δ*aprE*::lox72, Δ*nprE*::lox72, Δ*vpr*::lox72, Δ*epr*::lox72, Δ*wprA*::lox72	This study	***wprA***
*Bs*6051 LS8P-D	Δ*sacA*::SpecR, ΔRM::lox72, Δ*lytC*::lox72, Δ*bpr-spo*::lox72, Δ*nprB*::lox72, Δ*mpr*::lox72, Δ*aprE*::lox72, Δ*nprE*::lox72, Δ*vpr*::lox72, Δ*epr*::lox72, Δ*wprA*::lox72	This study	***cre* cassette**
*Bs*6051 LS8P-D Δ*amyE*	*Bs*JK49 with Δ*sacA*::SpecR, ΔRM::lox72, Δ*lytC*::lox72, Δ*bpr-spo*::lox72, Δ*nprB*::lox72, Δ*mpr*::lox72, Δ*aprE*::lox72, Δ*nprE*::lox72, Δ*vpr*::lox72, Δ*epr*::lox72, Δ*wprA*::lox72, Δ*amyE*::lox72	This study	***amyE***
*Bs*6051 LS8P-D Δ*amyE,* Δ*sacA*	Δ*sacA*::SpecR, ΔRM::lox72, Δ*lytC*::lox72, Δ*bpr-spo*::lox72, Δ*nprB*::lox72, Δ*mpr*::lox72, Δ*aprE*::lox72, Δ*nprE*::lox72, Δ*vpr*::lox72, Δ*epr*::lox72, Δ*wprA*::lox72, Δ*amyE*::lox72	This study	***cre* cassette**
*Bs*WB600 pSox	pMSE3-P_*acoA*_-Sox	This study	***-—-***
*Bs*6051 LS8P-D Δ*amyE* pSox	pMSE3-P_*acoA*_-Sox	This study	***-—-***
*Bs*JK135	*Bs*JK49 with Δ*lytC*::lox72, Δ*bpr*-*spo*::lox72, Δ*nprB*::lox72, Δ*mpr*::lox72, Δ*aprE*::lox72, Δ*nprE*::lox72, Δ*vpr*::lox72, Δ*epr*::lox72, Δ*wprA*::lox72, Δ*srfA*::lox72	This study	***sfrA***
*Bs*JK136	*Bs*JK49 with Δ*lytC*::lox72, Δ*bpr*-*spo*::lox72, Δ*nprB*::lox72, Δ*mpr*::lox72, Δ*aprE*::lox72, Δ*nprE*::lox72, Δ*vpr*::lox72, Δ*epr*::lox72, Δ*wprA*::lox72, Δ*srfA*::lox72, Δ*pksX*::lox72	This study	***pksX***
*Bs*JK137	*Bs*JK49 with Δ*lytC*::lox72, Δ*bpr*-*spo*::lox72, Δ*nprB*::lox72, Δ*mpr*::lox72, Δ*aprE*::lox72, Δ*nprE*::lox72, Δ*vpr*::lox72, Δ*epr*::lox72, Δ*wprA*::lox72, Δ*srfA*::lox72, Δ*pksX*::lox72, Δ*pps*::lox72	This study	***pps***
*Bs*JK138	*Bs*JK49 with Δ*lytC*::lox72, Δ*bpr*-*spo*::lox72, Δ*nprB*::lox72, Δ*mpr*::lox72, Δ*aprE*::lox72, Δ*nprE*::lox72, Δ*vpr*::lox72, Δ*epr*::lox72, Δ*wprA*::lox72, Δ*srfA*::lox72, Δ*pksX*::lox72, Δ*pps*::lox72, Δ*amyE*::lox72	This study	***amyE***
*Bs*JK139	Δ*sacA*::SpecR, ΔRM::lox72, Δ*lytC*::lox72, Δ*bpr*-*spo*::lox72, Δ*nprB*::lox72, Δ*mpr*::lox72, Δ*aprE*::lox72, Δ*nprE*::lox72, Δ*vpr*::lox72, Δ*epr*::lox72, Δ*wprA*::lox72, Δ*srfA*::lox72, Δ*pksX*::lox72, Δ*pps*::lox72, Δ*amyE*::lox72	This study	***cre* cassette**

**Table 2:** Overview of relevant plasmids for the construction of the *B. subtilis* strains investigated in this study

Plasmid	Relevant features	Reference	Relevant application
pUC19	*E. coli* plasmid	Schweizer (1991)	**Backbone for cloning procedures**
pMSE3	*B. subtilis* shuttle vector	Silbersack et al. (2006)	**High-copy expression vector**
pBE-S	*B. subtilis* shuttle vector	Takara	**Medium-copy expression vector**
pHB201	*B. subtilis* shuttle vector	Bron et al. (1998)	**Low-copy expression vector**
pJET-lox-SSS	Contains lox-SSS cassette (lox71-six-site-SpecR-six-site-lox66)	Kumpfmüller et al. (2013)	**DNA template**
pUC19-lox-SSS	Contains lox-SSS cassette isolated from pJET-lox-SSS	This study	
pUC19-lox-SSS-FB-*lytC*	Contains lox-SSS cassette and *lytC*-landing pads (*lytC*-front; *lytC*-back)	This study	***lytC *knockout**
pUC19-lox-SSS-FB-*bpr-spo*	Contains lox-SSS cassette and *bpr-spo* landing pads (*bpr*-front; *spoIIGA*-back)	This study	***bpr-spo* knockout**
pUC19-lox-SSS-FB-*nprB*	Contains lox-SSS cassette and *nprB* landing pads (*nprB*-front; *nprB*-back)	This study	***nprB* knockout**
pUC19-lox-SSS-FB-*mpr*	Contains lox-SSS cassette and *mpr* landing pads (*mpr*-front; *mpr*-back)	This study	***mpr* knockout**
pUC19-lox-SSS-FB-*aprE*	Contains lox-SSS cassette and *aprE* landing pads (*aprE*-front; *aprE*-back)	This study	***aprE* knockout**
pUC19-lox-SSS-FB-*nprE*	Contains lox-SSS cassette and *nprE* landing pads (*nprE-*front; *nprE*-back)	This study	***nprE* knockout**
pUC19-lox-SSS-FB-*vpr*	Contains lox-SSS cassette and *vpr* landing pads (*vpr*-front; *vpr*-back)	This study	***vpr* knockout**
pUC19-lox-SSS-FB-*epr*	Contains lox-SSS cassette and *epr* landing pads (*epr*-front; *epr*-back)	This study	***epr* knockout**
pUC19-lox-SSS-FB-*wprA*	Contains lox-SSS cassette and *wprA* landing pads (*wprA*-front; *wprA*-back)	This study	***wprA* knockout**
pJK191	Contains lox-SSS cassette and *srfA* landing pads (*srfA*-front; *srfA*-back)	Kumpfmüller et al. (2016)	***srfA* knockout**
pJK179	Contains lox-SSS cassette and *pksX* landing pads (*pksX*-front; *pksX*-back)	Kumpfmüller et al. (2016)	***pksX* knockout**
pJK254	Contains lox-SSS cassette and *pps* landing pads (*pps*-front; *pps*-back)	Kumpfmüller et al. (2016)	***pps* knockout**
pJK196	Integration of *comS* operon (*P*_*spac*_) and *cre* operon (*P*_*xylA*_) into *sacA* locus with Zeo^R^	Zobel et al. (2015)	**Integration of *cre* operon**
pJK226	Deletion of restriction and modification system	Zobel et al. (2015)	**RMS knockout**
pJK256	Contains *sacA* landing pads (*sacA*-front; *sacA*-back) and spectinomycin marker	Zobel et al. (2015)	**Removal of *cre-*marker cassette**
pAMY-lox-SSS	Contains lox-SSS cassette and *amyE* landing pads (*amyE*-front; *amyE*-back)	Kumpfmüller et al. (2013)	***amyE* knockout**
pSC-B-*bpr-spo*-lox-SSS	Contains lox-SSS cassette and *bpr-spo* landing pads (*bpr*-front; *spoIIGA*-back)	This study	**PCR template**
pSC-B-*mpr*-lox-SSS	Contains lox-SSS cassette and *mpr* landing pads (*mpr*-front; *mpr*-back)	This study	**PCR template**
pSC-B-*wprA*-lox-SSS	Contains lox-SSS cassette and *wprA* landing pads (*wprA*-front; *wprA*-back)	This study	**PCR template**
pSox	High-copy expression vector for P_*acoA*_-regulated Sox production	This study	***sox* expression, PCR template**
pUC19-IL1B-co	pUC19 vector with codon optimized IL1B sequence	This study/Genscript	**PCR template**

All routine molecular biological techniques were carried out according to standard protocols (Sambrook and Russell 2001). Restriction enzymes and other DNA-modifying enzymes were used as specified by the suppliers (Roche, Mannheim, Germany; New England Biolabs, Frankfurt a. M., Germany). DNA sequencing was carried out by Eurofins Genomics (Ebersberg, Germany). Oligonucleotides were synthesized and provided by Life Technologies (Darmstadt, Germany).

### *Plasmid and strain constructions*

#### Construction of B. subtilis LS8P-D

In order to construct an optimized *B. subtilis* expression strain, which is deficient in the eight main extracellular proteases and shows reduced autolysis as well as sporulation rates, the respective genes were deleted from the host genome using the Cre-*loxP* recombination technique as described by Kumpfmüller et al. (Kumpfmüller et al. 2013, 2016). For this purpose, *B. subtilis* strain *Bs*JK49, carrying a P_*xyl*_*-cre*, *xylR*, P_*spac*_-*comS*, ZeoR cassette in the *sacA* locus for induction of the Cre recombinase, was used as the host strain for all further modifications. The vector backbone of pUC19 (Schweizer 1991) was used for all cloning procedures.

#### Deletion of the native lytC gene

For deletion of the native *lytC* gene of the *B. subtilis* host strain, the lox-SSS cassette was isolated from pJET-loxSSS (Kumpfmüller et al. 2013) and gel-purified using the Qiaquick Gel Extraction Kit (Qiagen, Hilden, Germany). The purified lox-SSS fragment was then ligated with the *Xba*I- and *Bam*HI-restricted vector pUC19, and *E. coli* DH10B was transformed with the resulting recombinant plasmid, designated pUC19-loxSSS. The *lytC*-landing pads were amplified by PCR with oligonucleotides SO33/SO34 (*lytC*-front) and SO35/SO36 (*lytC*-back) using genomic DNA of *B. subtilis* as template. The *lytC*-front fragment was digested with *Sph*I/*Xba*I and ligated with the *Sph*I/*Xba*I-digested vector pUC19-lox-SSS. The recombinant plasmid was used for transformation of *E. coli* DH10B, yielding pUC19-lox-SSS-*lytC*F. Subsequently, the *Bam*HI/*Kpn*I digested and purified *lytC*-back fragment was ligated with pUC19-lox-SSS-*lytC*F. The recombinant plasmid was used for transformation of *E. coli* DH10B and sequence identity of all three DNA fragments was validated. The resulting plasmid was designated pUC19-lox-SSS-FB-*lytC*. Chromosomal integration of the *lytC* knockout vector and subsequent recombination of the lox sites was carried out as described before (Kumpfmüller et al. 2013). The resulting strain was designated *Bs*JK49 *ΔlytC* (Table 1).

#### Deletion of the spoIIGA and bpr genes

The deletion of the chromosomal copies of the *B. subtilis spoII*GA and *bpr* genes was combined in one step since both genes are located immediately adjacent to each other within the host genome. The respective DNA fragment, comprising the lox-SSS cassette and both landing pads, was amplified from pSC-B-*bpr*-*spo*-lox-SSS (Table 2) using oligonucleotides SO21 and SO22. The corresponding PCR product was digested, gel-purified, and integrated into the *Bam*HI and *Xba*I sites of pUC19. *Escherichia coli* DH10B was transformed with the recombinant plasmid pUC19-lox-SSS-FB-*bpr-spo* and sequence identity was validated. After transformation of *Bs*JK49 Δ*lytC*, strain *Bs*JK49 Δ*lytC*, ΔPI was obtained.

#### Deletion of the nprB, mpr, aprE, nprE, vpr, epr, and wprA genes

For *nprB*, *epr*, *vpr*, *aprE*, and *nprE*, the knockout of the genome-encoded protease genes was carried out by integration of the respective gene-specific upstream and downstream sequences in the vector backbone of pUC19-lox-SSS. The front regions of the aforementioned protease genes were amplified with oligonucleotides SO38/SO39 (*epr*), SO42/SO43 (*nprB*), SO46/SO47 (*nprE*), SO50/SO51 (*aprE*), and SO56/SO57 (*vpr*). After restriction with *Kpn*I/*Bam*HI and subsequent PCR product purification, all upstream fragments were ligated with the *Kpn*I- and *Bam*HI-restricted plasmid pUC19-lox-SSS. *Escherichia coli* DH10B was transformed with the resulting intermediate plasmids carrying the lox-SSS cassette and the respective upstream region. Amplification of the downstream regions was carried out using oligonucleotides SO40/SO41 (*epr*), SO44/SO45 (*nprB*), SO48/SO49 (*nprE*), SO52/SO53 (*aprE*), and SO58/SO59 (*vpr*). The *Xba*I/*Sph*I digested and purified DNA fragments were ligated with the corresponding intermediate plasmid. *Escherichia coli* DH10B was transformed with the resulting plasmids, designated pUC19-lox-SSS-FB-*epr*, pUC19-lox-SSS-FB-*nprB*, pUC19-lox-SSS-FB-*nprE,* pUC19-lox-SSS-FB-*aprE*, and pUC19-lox-SSS-FB-*vpr*. For *mpr* and *wprA* deletions, the appropriate DNA fragments comprising the lox-SSS cassette as well as the upstream and downstream regions of either *mpr* or *wprA* were amplified from pSC-B-*mpr*-lox-SSS (using oligonucleotides SO29/SO30) and pSC-B-*wprA*-lox-SSS (with oligonucleotides SO17/SO18), respectively. The purified and *Bam*HI/*Xba*I-digested DNA fragments were then separately ligated with the *Xba*I- and *Bam*HI-restricted vector pUC19. *Escherichia coli* DH10B was transformed with the resulting recombinant plasmids, designated pUC19-lox-SSS-FB-*mpr* and pUC19-lox-SSS-FB-*wprA*. Sequence identity of all pUC19-knockout vectors was validated, and transformation of *Bs*JK49 Δ*lytC*, ΔPI was carried out successively. The final strain, *B. subtilis* LS8P-D, deficient in eight proteases and characterized by reduced autolysis and sporulation rates was obtained by replacing the Cre-ZeoR-cassette in the *sacA* locus of *Bs*JK49 Δ*lytC*, ΔPVIII with the sequence of the spectinomycin selection marker. To this purpose, the integrative plasmid pJK256 (Zobel et al. 2015) was used. All gene deletions in the final *B. subtilis* LS8P-D strain as well as in all intermediate strains were confirmed by PCR in comparison to the unmodified parental strain *Bs*JK49 and subsequent sequencing of the PCR products.

#### Deletion of secondary metabolite gene clusters

Further modification of the *B. subtilis* LS8P-D precursor strain *Bs*JK49 Δ*lytC*, ΔPVIII was carried out by deletion of three big gene clusters for major secondary metabolite production. First the *srfA* cluster, responsible for the biosynthesis of surfactin was deleted using pJK191 (Kumpfmüller et al. 2016) (Table 2). This vector contained the lox-SSS cassette flanked by the homology regions for the *srfA* cluster. After transformation of *Bs*JK49 Δ*lytC*, ΔPVIII, and subsequent induction of Cre recombinase, *srfA* deletion was confirmed by PCR and sequencing. The resulting strain was designated *Bs*JK135. The subsequent deletions of the *pksX* gene cluster for bacillaene biosynthesis and the *pps* cluster for pliplastatin biosynthesis were carried out with pJK179 and pJK254 (Kumpfmüller et al. 2016) respectively. One after the other, the deletion vectors were used for transformation of *Bs*JK135 (Δ*srfA*) and *Bs*JK136 (Δ*srfA*, Δ*pksX*), yielding strain *Bs*JK137 (Δ*srfA*, Δ*pksX*, Δ*pps*). All deletions were verified by PCR and sequencing. Additionally, to ensure cultivation in EnpressoB medium, the amylase gene in *Bs*JK137 was deleted by transformation of the strain with plasmid pAMY-lox-SSS (Kumpfmüller et al. 2013), and the resulting strain was designated *Bs*JK138. Finally, the Cre cassette was removed from the genome of *Bs*JK138 using pJK256 as described above. This further evolved strain, *Bs*JK139, was verified by PCR. The relevant genotypes of *Bs*JK139 and all intermediate strains are listed in Table 1.

#### Construction of the plasmid library for Sox activity screening

In order to obtain a *sox* plasmid library with 173 different types of signal peptide (SP) DNA sequences in the required size of at least 2000 *E. coli* clones, the “*B. subtilis* Secretory Protein Expression System” (Takara/Clontech) in combination with the “In Fusion HD Cloning Plus Kit” (Takara/Clontech) was used according to the manufacturer’s instructions. To this end, the *sox* gene was amplified from pSox (Table 2) using oligonucleotides TB20 and TB21. After restriction with *Nde*I and *Xba*I, the purified PCR product and the pBE-S vector were ligated and *E. coli* DH10B was transformed with the recombinant plasmid pBE-S-*sox*. After validation of sequence identity, all different signal peptide sequences included in the provided SP library were integrated into the vector backbone of pBE-S-*sox* following the manufacturer’s instructions. In brief, the *Eag*I- and *Mlu*I-digested vector was ligated with the 173 SP-containing DNA mixture using the “In Fusion Cloning” technology. Chemically competent *E. coli* Stellar cells (included in the kit) were transformed with 2 μL of the “In Fusion” reaction and selected on LB agar plates with ampicillin. All colony-forming units (cfu) were rinsed from the plate to isolate the *sox* plasmid library, which was subsequently integrated into the expression host *B. subtilis* LS8P-D Δ*amyE* by electroporation.

### Cultivation conditions and media

Sox expression-prescreening experiments (Fig. S2) were carried out in 48-well plates (1-mL scale). Production strains that were identified in the screening experiments were then cultivated in 24-well deep-well plates for comparative *sox* expression (3.5-mL scale). For this purpose, EnpressoB medium was used for the expression studies with the host strain *B. subtilis* LS8P-D Δ*amyE* following the manufacturer’s instructions. Briefly, for each cultivation, an LB pre-culture was grown for 6–8 h at 250 rpm and 37 °C and was subsequently used to inoculate the main culture. The main culture was grown at 30 °C and 37 °C for 15–18 h. The booster solution was added in a 1:10 ratio and growth was continued for another 2 days. Cell harvesting was carried out 24 h and 48 h after addition of the booster solution. In case of all P_*acoA*_-regulated expression systems, the addition of the inducer acetoin (final concentration 1%) was necessary and occurred simultaneously with the addition of the booster solution. All cultivation and protein assay experiments were performed with at least three biological replicates.

### Protein purification

For Sox activity screening, His-tag-based purification was realized using the HisSorb Plates (Qiagen) as recommended by the manufacturer. To this end, 200 μL of cell supernatant was bound to the plate surface, washed, and eluted in a final volume of 50 μL.

For determination of enzymatic activities derived from the cultivations in 3.5-mL and 1.5-L scale, ProCatch His Resin (Miltenyi Biotec) and Roti®garose-His/Ni Beads (Carl Roth) were used following the manufacturers’ instructions. In brief, 1 mL of the culture supernatant was incubated with 500 μL of His Resin for at least 20 min. After protein binding and washing procedures, the purified Sox protein was eluted from the His Resin with 200 μL elution buffer and was subsequently used for activity measurements.

### Protein analysis

SDS-PAGE was carried out as described by Laemmli (Laemmli 1970). Four percent stacking gels and 15% or 18% separating gels were used depending on the size of the detected protein. Protein samples (20 μL) were mixed with 4 μL of 4 × sample buffer, denatured for 10 min at 95 °C, and loaded on the gel. SDS gel staining was carried out using Coomassie staining solution.

### Protein quantification

If protein amounts are quantified from the culture supernatant, no dilution or concentration steps can impair the results. Thus, a realistic condition is depicted. For protein quantification, the gels were consistently loaded with all triplicate samples from the biological replicates, a molecular weight standard, and four dilutions of a BSA standard with known concentrations of 25, 50, 100, and 200 μg/mL. Based on the calibration curve derived from the BSA standard, the signal intensities of all protein samples were converted into protein amounts. To this end, the GelAnalyzer 2010 software was used. Based on the obtained data, the amounts of Sox and interleukin derived from all 12 expression strains were calculated and compared.

## Results

The production of oxidative enzymes like the sulfhydryl oxidase Sox represents a challenge. These enzymes are able to produce reactive oxygen species as part of their catalyzed enzymatic reaction, which lead to damages of host cell components and concomitant low enzyme yields. Besides sophisticated and expensive small-scale cell-free production of these potential toxic proteins, secretion of the oxidative target enzymes to the medium was found to be one option to overcome part of the toxicity problem. Thus, *Bacillus* strains enabling extracellular protein accumulation are promising host candidates for the production of such oxidative enzymes for biotechnological applications. We therefore tested *B. subtilis* as a secretory expression host for the synthesis of the sulfhydryl oxidase Sox.

### Development of a vector toolbox for B. subtilis

**Fig. 1:**
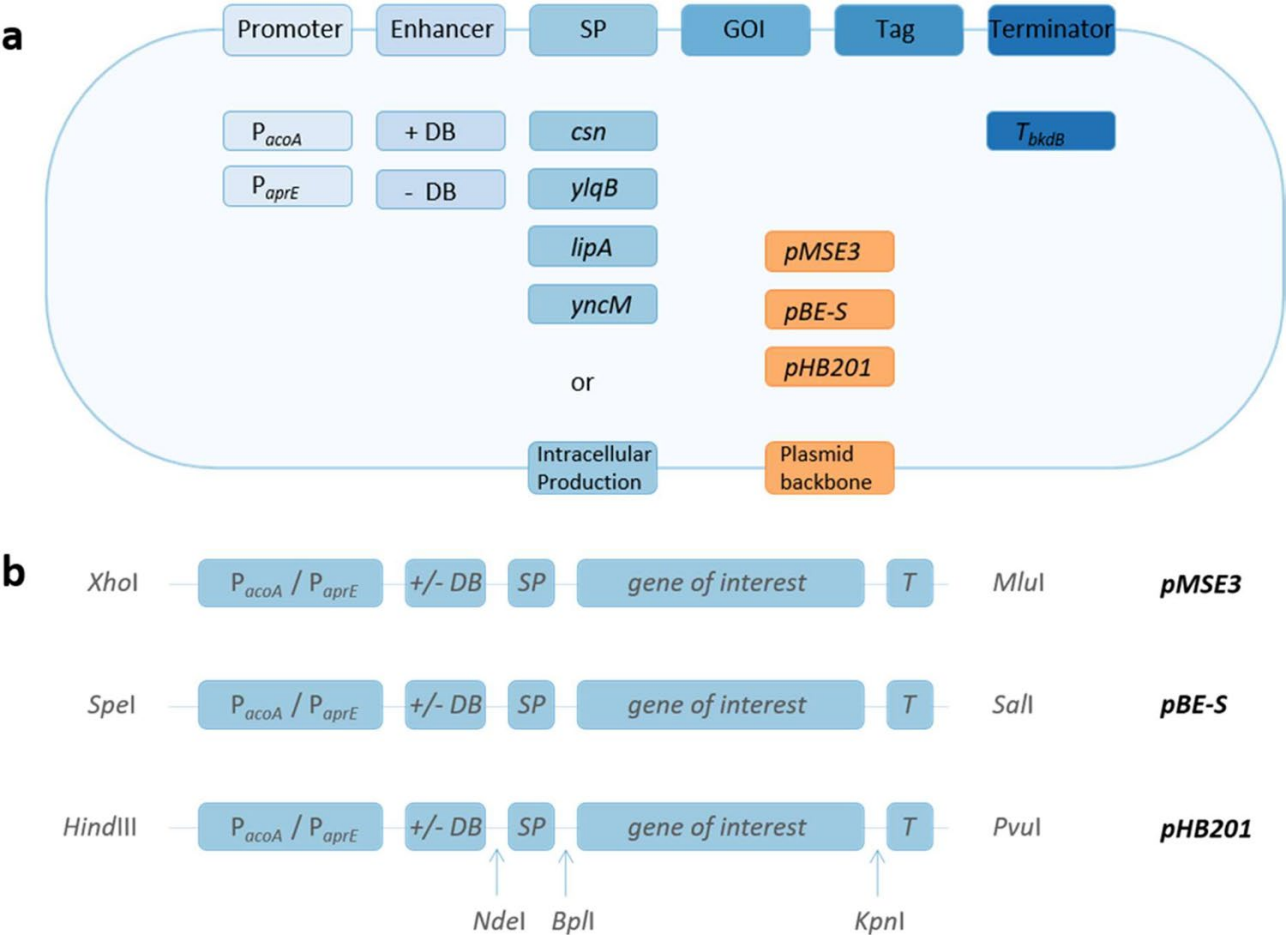
Schematic overview (**a**) of the replaceable elements of the *B. subtilis* toolbox and (**b**) the array of the toolbox elements including restriction sites. The multiple cloning site of all toolbox vectors was adjusted as shown in Figure S1. GOI, gene of interest

A successful secretory overexpression of foreign proteins, especially of eukaryotic origin, in bacterial expression systems cannot be coherently performed based on one standardized expression system alone. Due to the singularity of each individual heterologous protein, different host-vector systems and expression strategies have to be tested for optimal secretory overproduction. Therefore, we developed a vector toolbox for *B. subtilis*, which combines different strong secretion signals, vector backbones for the regulation of the gene copy number, two different inducible promoters, and a translational enhancer and a strong terminator sequence (Fig. 1). The construction of the plasmids in the toolbox is described in the S*upplementary Online Material* of this study. As backbone for this new host-vector system, we constructed the optimized *B. subtilis* expression strain LS8P-D, which is sporulation deficient and contains markerless gene deletions for eight main extracellular proteases (Table 1). After inactivation of the *lytC* gene, this strain shows reduced cell autolysis (Kabisch et al. 2013). Since potential bioactive compounds could be problematic in selected bioprocesses or in the application of overproduced enzymes, this strain was further modified by the deletion of three gene clusters responsible for the formation of the prominent secondary metabolites surfactin, bacillaene, and pliplastatin. The resulting strain *B. subtilis* BsJK139 was used in all experiments for the evaluation of the toolbox vectors constructed in this study.

**Fig. 2:**
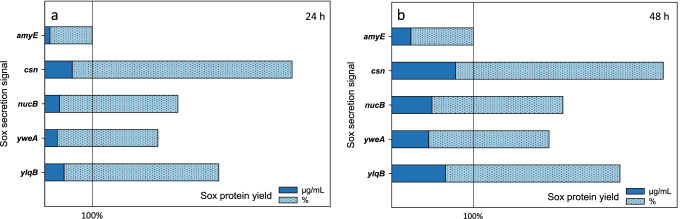
Sox amounts from SP screening. High amounts of Sox protein after 24 h (**a**) and 48 h (**b**) were calculated for the secretion signals of *csn*, *nucB*, *yweA*, and *ylqB* in comparison to significantly reduced amounts of Sox secreted by the *amyE* signal peptide. Dark blue bars represent Sox yields in μg/mL based on the quantification using a BSA standard (see Fig. S3b) while light blue bars illustrate the percentage distribution using the *amyE*-SP as reference (100%)

As a first step in the development of the toolbox, screening experiments were performed in order to determine suitable strong peptide secretion signals for efficient Sox export (see *Supplementary Online Material*). Highest yields of secreted Sox proteins were observed using the signal peptides of Csn, NucB, YweA, and YlqB (Fig. 2, Fig. S2-S3). The calculated protein amounts significantly exceeded those obtained with the previously used *amyE* signal peptide up to fivefold (Fig. 2). The secretion signals of *csn* and *ylqB*, which mediated the largest Sox quantities were thus used for the toolbox setup.

In addition, two further strong secretion signals deduced from the *B. subtilis lipA* and *yncM* genes were chosen for the toolbox based on literature data (Brockmeier et al. 2006). The toolbox enables modulation of the gene copy number by three vector backbones with high (pMSE3: 200–250 copies) (Silbersack et al. 2006), medium (pBE-S: 50 copies) (Leonhardt 1990), and low (pHB201: 5 copies) (Bron et al. 1987, 1998) copy numbers. Furthermore, the toolbox is based on the transient phase induced promoter (P_*aprE*_) of the *B. subtilis* subtilisin-encoding *aprE* gene (Veening et al. 2008) and the strictly regulated acetoin-inducible promoter (P_*acoA*_) of the *B. subtilis* acetoin dehydrogenase-encoding *acoABCD* operon (Silbersack et al. 2006). In addition, the downstream box (DB) of the *B. subtilis cspB* gene was included as a potential translational enhancer (Welsch et al. 2015). The strong transcriptional terminator structure derived from the *B. subtilis bkd* operon (Nickel et al. 2004) was chosen to prevent a potential transcriptional read through by the two strong promoters (Fig. 1a). Together with the possibility of intracellular protein production, this newly constructed toolbox consists of 60 vector variants (Table S2), each of which offers the possibility to easily exchange every single module by Gibson cloning or classical restriction and ligation (Fig. 1b).

### Verification of the toolbox by Sox production

**Fig. 3:**
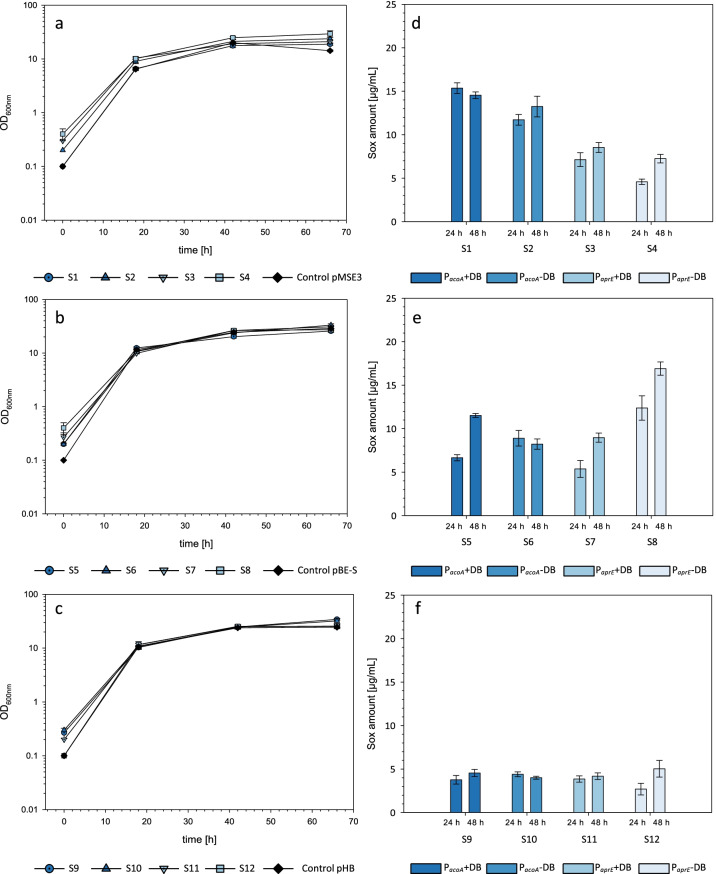
Verification of the toolbox strains S1–S12 (see also Table 3) for the overexpression of Sox. **a**–**c** Growth curves of *B. subtilis* JK138 S1–12 in comparison to the negative control carrying the “empty vector” without expression cassette. Cultivations were performed in biological triplicates; standard deviations are presented as error bars. **d**–**f** Quantitative analyses of the secretory overproduction of Sox. Average protein amounts (normalized by OD) for the Sox strains S1–S12 in the culture supernatant 24 h and 48 h after the boost in EnpressoB medium. Protein concentrations are given as average values from three independent experiments. Standard deviations are presented as error bars. For comparison of protein yields, the target protein was quantified. “Empty vector” strains without expression cassette served as negative controls

Using the sulfhydryl oxidase Sox (Lee et al. 2000) of *S. cerevisiae* and the human interleukin-1β (IL) (Garlanda et al. 2013) as model heterologous proteins, the suitability of the vector toolbox for *B. subtilis* was evaluated. To gain an overview about the influence of different toolbox modules on the yield of Sox, a set of 12 expression strains (see Table 3) for this target protein was analyzed under small-scale conditions in EnpressoB medium (Fig. 3a–c). Our signal peptide prescreening experiments indicated that most efficient Sox secretion was mediated by the *csn* signal peptide. Consequently, this specific SP was used for the construction of the individual Sox-producing strains based on the toolbox (Table 3, Table S2). The generated toolbox strains 1–4 carry the high-copy vector backbone pMSE3, strains 5–8 the medium-copy backbone pBE-S, and strains 9–12 the low-copy vector pHB201. The four variants of each backbone differ in the promoter sequences (P_*acoA*_ for the numbers 1, 2, 5, 6, 9, 10, and P_*aprE*_ for the remaining 3, 4, 7, 8, 11, 12 strains) and in the presence (uneven numbers) or absence (even numbers) of the DB enhancer sequence (see Table 3)*.*

**Table 3:** Genetic variations of the toolbox-based *B. subtilis* strains for the secretory overexpression of the Sox (left) and IL (right) encoding genes. *SP*, signal peptide; *DB*, downstream box

Strain	Target gene + SP	Vector backbone	Promoter	DB	Strain	Target gene + SP	Vector backbone	Promoter	DB
S1	Sulfhydryl oxidase + *csn*	pMSE3	p_a*coA*_	+	IL1	Interleukin^1β^ + *lipA*	pMSE3	p_*acoA*_	+
S2				−	IL2				−
S3			p_*aprE*_	+	IL3			p_*aprE*_	+
S4				−	IL4				−
S5		pBE-S	p_*acoA*_	+	IL5		pBE-S	p_*acoA*_	+
S6				−	IL6				−
S7			p_*aprE*_	+	IL7			p_*aprE*_	+
S8				−	IL8				−
S9		pHB201	p_*acoA*_	+	IL9		pHB201	p_*acoA*_	+
S10				−	IL10				−
S11			p_*aprE*_	+	IL11			p_*aprE*_	+
S12				−	IL12				−

Sox quantification from the culture supernatants revealed pronounced differences in *sox* expression of the different vector setups, especially 24 h after the boost (Fig. 3d–f). For the pMSE3-carrying strains, highest Sox amounts (~ 12–15 μg/mL) were calculated for the P_*acoA*_-based expression strains (S1, S2), whereas significantly lower concentrations of approx. 5–7 μg/mL were calculated for the P_*aprE*_-based expression strains S3 and S4. The enhancer sequence in the strains S1 and S3 had a noticeable effect on protein production in comparison to their DB-free analogues S2 and S4. In contrast, within the pBE-S backbone, the absence of the enhancer sequence in S6 and S8 resulted in higher protein amounts compared to the analogous strains without DB (S5, S7) at t_24h_. In this background, highest secreted protein amounts were determined for S8 with 12.4 μg/mL. With average values of approximately 4 μg/mL, all low-copy vector-based expression strains S9-12 exhibited comparable but lower Sox yields. Forty-eight hours after the boost (Fig. 3d), the S1 and S2 strains still reached higher Sox concentrations compared to S3 and S4. However, slightly increased values were calculated for the strain variants without the enhancer sequence. The pBE-S-based strains S5-7 (Fig. 3e) showed similar protein amounts of about 10 μg/mL, whereas the Sox concentration for strain S8 without the enhancer sequence reached up to 17 μg/mL. The lowest protein amounts (approx. 5 μg/mL) were again determined for the S9-S12 strains which harbor the low-copy expression vectors (Fig. 3f). Thus, the highest *sox* expression was constantly reached by the high-copy vector with the acetoin-controlled P_*acoA*_ promoter in strain S1 (Fig. 3d).

The toolbox was also evaluated by the overproduction of the human interleukin-1β (IL) (see *Supplementary Online Material*). The experimental setup for this second model protein was equal to the *sox* expression procedures. It is shown that the IL constructs gave similar results as the *sox* toolbox strains (Fig. S4). The highest IL production was reached with the IL2 toolbox setup containing the high-copy vector and the acetoin-controlled P_*acoA*_ promoter.

### Determination of Sox activity

**Fig. 4:**
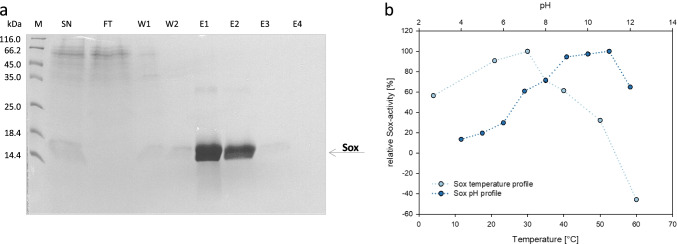
Activity of the heterologously produced sulfhydryl oxidase Sox. **a** Purification of Sox from the supernatant of *B. subtilis* WB600 pSox cultures; SN, supernatant; FT, flow through, W1–W2: wash fractions, E1–E4: elution fractions. **b** Temperature and pH profiles of purified Sox. The influence of temperature (light blue dots) and pH (dark blue dots) on Sox activity was determined using a miniaturized Sox assay in a semi-automated system applying a Tecan Freedom EVO® screening robot

In order to determine a functional expression of the sulfhydryl oxidase we established a suitable procedure for Sox activity analyses. Culture supernatants of the model strain *B. subtilis* WB600 pSox were concentrated by lyophilization and subsequent resuspension of the extracellular protein extracts in water. The pH of the obtained protein solution was adjusted to 8.0 and used for His-tag-based affinity chromatography. Sox purification from the supernatant was monitored by SDS-PAGE (Fig. 4a). Purified Sox proteins were used to determine temperature and pH profiles of this heterologously produced enzyme (Fig. 4b). Maximum Sox activity was determined at 30 °C and at alkaline pH ranging from pH 9 to 11.

### Fermentation strategies for Sox production

Essential fermentation parameters for heterologous *sox* expression in *B. subtilis* were identified and stepwise optimized. The small-scale cultivations indicated that the P_*acoA*_ promoter was most suitable for Sox (Fig. 3) as well as IL (Fig. S4) production. Therefore, we used this acetoin-inducible promoter system to develop a fermentation strategy for the Sox target protein. We investigated the influence of 30 °C vs. 37 °C as growth temperature on enzyme yield with this promoter system (Fig. S5). Although similar cell densities were measured after 12 h of cultivation, a faster growth in combination with an earlier start of Sox production was observed at 37 °C. We therefore performed all following fermentations at 37 °C. Furthermore, we tested the influence of different glucose concentrations on growth and productivity of *B. subtilis* WB600 pSox (Fig. S6). Our analyses revealed that both investigated glucose concentrations enabled similar Sox activities and final optical densities.

**Fig. 5:**
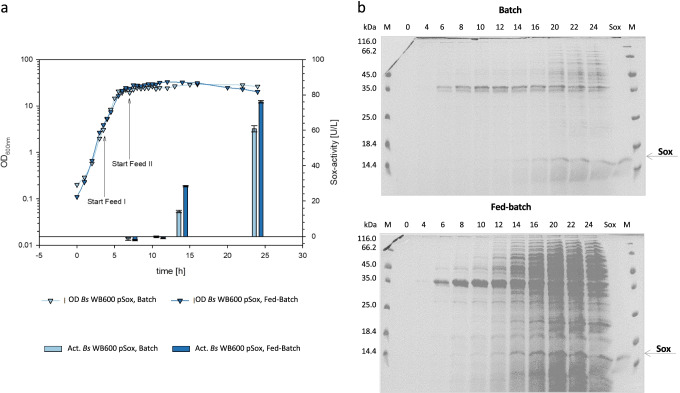
Development of a fermentation strategy for Sox overproduction. **a** Comparison of batch (light blue) and fed-batch (dark blue) fermentation strategies for Sox production in *B. subtilis* WB600 pSox. The cells were grown in a parallel fermenter system at 1.5 L scale at 37 °C either in batch or in fed-batch mode. Cell growth was monitored by the OD at 600 nm (triangles). Sox activity was determined in triplicates using purified enzyme (bars). **b** SDS-PAGE analysis of Sox accumulation in untreated culture supernatants of the batch and fed-batch fermentations

We therefore used these temperature and glucose parameters to develop a fed-batch fermentation strategy, which was compared to the established batch process (Fig. 5a). The batch fermentation was performed again with NBMM containing threefold concentrated complex components yeast extract and peptone (45 and 9.6 g/L, respectively), 0.5% (w/v) acetoin and 10 g/L glucose. The fed-batch fermentation was carried out with a starting batch containing NBMM medium with 15 g/L yeast extract, 3.2 g/L peptone, 0.5% (w/v) acetoin, and 10 g/L glucose. Feed I was started at 4-h fermentation time with 7.5 g/h yeast extract, 1.6 g/h peptone, and 4.0 g/h glucose. Feed II was started at 7-h fermentation time with 7.5 g/h yeast extract and 1.6 g/h peptone. Growth curves and Sox activities were determined as before and showed that the consecutive feeding strategy applied in the fed-batch fermentation yielded maximum optical densities of 33.0 after 12 h compared to 29.4 after 16 h in the batch fermentation and seemed to result in higher autolysis rates. A significant increase in Sox activity in the fermentation supernatant of 76.1 U/L after 24 h was determined when applying a fed-batch rather than a batch fermentation strategy, which yielded 60.8 U/ L after 24 h (Fig. 5a). This was confirmed by SDS-PAGE analyses which revealed increased amounts of Sox protein during the fed-batch fermentation in the total extracellular protein extract. Thus, the fed-batch strategy enabled higher Sox activities and levels compared to the batch process (Fig. 5).

**Fig. 6:**
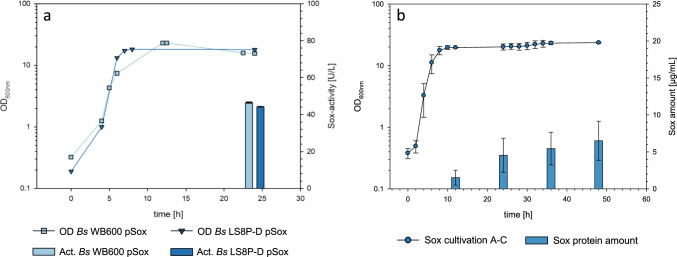
**a** Comparison of growth and Sox production of the protease-deficient *B. subtilis* strains WB600 (light blue) and LS8P-D (dark blue). The cells were grown in batch fermentations at 37 °C (triangles). Sox activity was determined in triplicates using purified enzyme after 24 h (bars). **b** Verification of the best performing P_*acoA*_-dependent S1 toolbox system in *B. subtilis* JK139 for the overexpression of the sulfhydryl oxidase Sox. The growth curve reflects three individual 1.5-L fed-batch fermentation experiments A–C. Shown are average protein amounts (normalized by OD) from the culture supernatant of the S1 expression strains from three independent experiments. Standard deviations are presented as error bars

The cultivation experiments with the protease-deficient *B. subtilis* strain WB600 revealed an autolysis of the cells throughout the fermentation, especially at 37 °C and during the stationary phase. For this reason, Sox production of *B. subtilis* WB600 pSox was compared with the novel tailor-made expression strain LS8P-D pSox, which possesses not only clean gene deletions of the eight major extracellular proteases and three active secondary metabolite clusters but which is also deficient in the *lytC* gene (Fig. 6a). In these experiments, the NBMM medium was supplemented with twofold concentrated yeast extract and peptone (30 and 6.4 g/L, respectively) and 2 g/L glucose. Since additional tests in small-scale showed that reduction of the inductor acetoin was possible without decrease in productivity (data not shown), only 0.05% (w/v) acetoin was used to induce Sox production. This medium composition resulted in a slightly higher final optical density of *B. subtilis* LS8P-D pSox compared to the WB600 pSox strain (Fig. 6a). This higher final optical density and the more stable stationary phase of the *B. subtilis* LS8P-D pSox cultivations indicated reduced autolysis of this novel strain. Furthermore, compared to the WB600 strain, the engineered LS8P-D expression strain revealed similar Sox activities in the extracellular protein supernatant (Fig. 6a).

We finally used this *B. subtilis* LS8P-D mutant background in form of the novel *B. subtilis* mutant strain *Bs*JK139 and the established large-scale fed-batch fermentation strategy to test the overproduction of the best performing Sox and IL toolbox setups. In both expression strains, the high-copy vector pMSE3 was used, and in the case of the Sox-producing setup, S1 the DB enhancer sequence was included. Each of the 1.5-L fed-batch cultivations for either Sox or IL production was carried out in triplicates. Samples for protein analyses were taken after 12, 24, 36, and 48 h (Fig. 6b, Fig. S7). Expression of the *sox* gene was induced by initial addition of 0.5% (w/v) acetoin. The calculated Sox amounts in the supernatant of all three fermentations showed a continuous increase in protein concentrations from 1.8 (t_12h_) to 6.5 μg/mL (t_48h_) over the whole fermentation time (Fig. 6b). Compared to the small-scale cultivations, however, slightly lower amounts of the Sox target protein were produced. We tested the same fed-batch fermentation strategy for the best performing IL producing toolbox setup IL2 in the novel *B. subtilis Bs*JK139 expression strain by using the newly established large-scale fed-batch strategy (Fig. S7). A similar expression pattern of the interleukin was determined in these cultivation experiments. However, in contrast to Sox production, comparable IL protein amounts were calculated in the small- and large-scale fed-batch cultivations.

## Discussion

The aim of this study was to develop a versatile expression platform for the secretory production of heterologous proteins in *B. subtilis*. The different copy numbers of the three vector backbones of the established toolbox enable a sensitive fine-tuning of target gene expression by affecting the amounts of transcripts which, in turn, determine the rate of target protein synthesis. However, high-copy numbers of plasmids can also negatively influence cell physiology due to the increased metabolic demands by plasmid DNA and mRNA synthesis as well as protein translation of encoded proteins. Such a cellular metabolic burden may lead to reduced capacities for target protein production or plasmid instabilities. Thus, a reasonable balance between plasmid copy number, expression strength of the chosen promoter, and recombinant protein synthesis is required for efficient protein production processes. We observed that the copy number of dependent expression of the two model genes of this study resulted in different expression rates by the toolbox vectors. Whereas the Sox protein reached highest secreted protein levels with the multi-copy vector backbone of pMSE3 or medium-copy plasmid pBE-S, the interleukin revealed rather weaker protein amounts with these high-copy variants but a slightly higher protein level with the low-copy vector pHB201.

Our toolbox analyses also indicated that the *acoA* promoter leads in most cases to a higher expression level of the two target genes compared to the *aprE* promoter. This observed trend for Sox overproduction might be partially due to the strict control of the *acoA* promoter which needs the inducer acetoin, but which is also only strongly active if the preferred carbon source glucose is exhausted (Silbersack et al. 2006). However, the *aprE* promoter that is controlled by DegU and induced during nutrient limitation (Veening et al. 2008) might also be competitive if an optimized fermentation strategy is used. Especially for the production of potential toxic enzymes, the P_*acoA*_ variants might be advantageous. A premature induction of the *acoA* promoter is repressed in the presence of glucose during exponential growth (Silbersack et al. 2006) and higher cell densities of the expression host prior to induction could be beneficial to ensure an efficient synthesis of critical proteins (Müller et al. 1996).

The in this study established toolbox (Table S2) with three different expression vector backbones, two promoters, four secretion signals, and the presence or absence of the translational enhancer sequence comprises 60 plasmids. Each module of this plasmid system can easily be exchanged by standard molecular cloning or assembly methods (Fig. 1b). This toolbox allows individual adjustments of heterologous protein expression and secretion, which was successfully evaluated by means of the two model proteins, sulfhydryl oxidase from *S. cerevisiae* and human interleukin-1β in this study.

Finally, the application of the novel toolbox is supported by the prototrophic protease and sporulation-deficient strain *Bs*JK139, which allows for improved downstream processing by reduced secondary metabolism and autolysis, and prevents premature proteolysis of target proteins. Although the obtained Sox yield is relatively low compared to other target proteins overproduced in *Bacilli* (Lakowitz et al. 2018), the combination of this engineered *B. subtilis* strain background with the tightly regulated *acoA* promoter and the established glucose- and acetoin-controlled fed-batch fermentation strategy enabled a significant overproduction of this potentially toxic sulfhydryl oxidase in an active form. In summary, the proposed expression toolbox in combination with the optimized host cell backbone ensures efficient recombinant production of proteins that are difficult-to-express in *B. subtilis*.

## Supplementary Information

Below is the link to the electronic supplementary material.

Supplementary file1 (PDF 1265 KB)

## Data Availability

The strains and plasmids constructed in this study as well as the data that support the findings of this study are available from the corresponding author upon request.
