# Supplementary material for: A host-vector toolbox for improved secretory protein overproduction in Bacillus subtilis
Source: Appl Microbiol Biotechnol. 2022 Jul 8;106(13-16):5137–51. doi: 10.1007/s00253-022-12062-2 (PMC9329435; doi:10.1007/s00253-022-12062-2)
Supplement: Supplementary file 1 — Supplementary file1 (PDF 1265 KB) [file 253_2022_12062_MOESM1_ESM.pdf]

Supporting information for:

Applied Microbiology and Biotechnology

A host-vector toolbox for improved secretory protein overproduction in *Bacillus subtilis*

Anna Krüger[1]*, Norma Welsch[2,3]*, Alexandra Dürwald[2], Henrike Brundiek[4], Rainer Wardenga[4], Henning Piascheck[1], Hendrik G. Mengers[5], Jana Krabbe[2,6], Sandra Beyer[2,7], Johannes F. Kabisch[2,8], Lutz Popper[9], Tanno Hübel[10], Garabed Antranikian[1], Thomas Schweder[2,3#]

[1]*Institute of Technical Microbiology, Hamburg University of Technology, Kasernenstr. 12, D-21073 Hamburg, Germany*

[2]*Pharmaceutical Biotechnology, Institute of Pharmacy, University of Greifswald, Felix-Hausdorff-Str. 3, D-17487 Greifswald, Germany*

[3]*Institute of Marine Biotechnology, Walther-Rathenau-Str. 49, D-17489 Greifswald, Germany*

[4]*Enzymicals AG, Walther-Rathenau-Straße 49a, D-17489, Greifswald, Germany*

[5]*Institute of Applied Microbiology - iAMB, Aachen Biology and Biotechnology - ABBt, RWTH Aachen University, Worringerweg 1, D-52074, Aachen, Germany*

[6]*Department of Biomolecular Chemistry, Leibniz Institute for Natural Product Research and Infection Biology, HKI, Beutenbergstr. 11a, D-07745, Jena, Germany*

[7]*Eppendorf AG, Bioprocess Center, Rudolf-Schulten-Str. 5, D-52428 Jülich, Germany*

[8]*Department of Biotechnology and Food Science, NTNU, Sem Sælands vei 6, 7034 Trondheim, Norway*

[9]*Stern Enzym GmbH & Co. KG, Kurt-Fischer-Str. 55, D-22926 Ahrensburg, Germany*

[10]*Miltenyi Biotec GmbH, Robert-Koch-Str. 1, D-17166 Teterow, Germany*

**Authors contributed equally*

[#]Corresponding author:

Thomas Schweder

Email: schweder@uni-greifswald.de

Tel.: +49 3834 420 4212

Fax: +49 3834 420 4238

This SOM part includes:

Material and Methods

Results

Tables S1, S2 and S3

Figures S1 to S7

References

Material and Methods

Construction of the pSox expression vector

The nucleotide sequence of the *sox* expression cassette, consisting of *acoA* promoter, *amyE* encoding signal peptide sequence, *sox* gene (Genbank Accession number MZ783064), His-Tag sequence and T7 terminator was synthesized and provided by GenScript Biotech (Leiden, Netherlands). For this purpose, the synthetic *sox* gene was codon-optimized for expression in *B. subtilis* using the GenSmart^{TM} Codon Optimization tool (GenScript). The algorithm utilizes a matrix for the most frequently occurring codons in *B. subtilis*. The construct was assembled from synthetic oligonucleotides and cloned into the pMSE3 (Silbersack et al. 2006) backbone using the *Eco*RI and *Xba*I restriction sites.

Construction of *Bs*JK49

*Bs*JK49 served as the host strain for all further modifications resulting in strains *B. subtilis* LS8P-D and *Bs*JK139. *Bs*JK49 was constructed by integration of pJK196 (Zobel et al. 2015), carrying the *cre*-operon, into the *sacA* locus of the *B. subtilis* ATCC6051HGW (Kabisch et al. 2013) genome. The resulting intermediate strain was designated *Bs*JK32. Subsequently, the restriction modification system (RMS) in *Bs*JK32 was deleted with plasmid pJK226 (Zobel et al. 2015), leading to *Bs*JK49.

Construction of the *B. subtilis* expression toolbox

For toolbox construction and easier handling, a fragment of a β-galactosidase gene from *Pseudoalteromonas haloplanktis* (Welsch et al. 2012) was incorporated into all vector backbones. This additional fragment enables the distinction between complete and incomplete vector digestion by *Bpl*I in restriction ligation reactions in order to increase transformation efficiency since *Bpl*I digestion is never complete. *Bpl*I was chosen for seamless cloning of the target genes since no additional foreign bases remain inside the translated protein sequence. However, to also offer a multiple cloning site within the toolbox vectors, the recognition sequence of *Bpl*I (Fig. S1a) was adjusted to this purpose and contains additional restriction sites for *Sac*I, *Nru*I, *Sph*I, *Nae*I and *Eag*I (Fig. S1b). For exchange of individual toolbox modules further unique recognition sites were identified (Fig. 1b). All toolbox vectors are listed in Table S2.

a NNNNN/NNNNNNNNGAGNNNNNCTCNNNNNNNNNNNNN/

/NNNNNNNNNNNNNCTCNNNNNGAGNNNNNNNN/NNNNN

b NNNNN/NNNNNNNNGAGNNNNNCTCNNNNNNNNNNNNN/

NNNNN/GAGCTCGCGAGCATGCCTCGCCGGCCGNNNNN/

*Sac*I GAGCTC | *Nru*I TCGCGA | *Sph*I GCATGC | *Nae*I GCCGGC | *Eag*I CGGCCG

Figure S1 Recognition sequence of restriction endonuclease *Bpl*I (**a**) and generation of the multiple cloning site by adjustment of the *Bpl*I recognition sequence (**b**).

Construction of toolbox vectors with csn signal peptide

All single elements for preparation of the basic toolbox vectors with *csn* secretion signal were successively cloned into the backbone of the high copy vector pMSE3. To this end, the *lacZ*-fragment was amplified by PCR using Opti Taq Polymerase (Roboklon, Berlin, Germany) with oligonucleotides TB76/TB77 and plasmid pTAE79 (Welsch et al. 2012) as the template. The amplicon served as template for the following PCR with oligonucleotides TB81/TB82, which fused the *bkdB* terminator and the *Kpn*I and *Mlu*I restriction sites to the 3´- terminus and the *Eag*I recognition sequence to the 5'- terminus of the PCR fragment. PCR product digestion with *Eag*I and *Mlu*I and subsequent purification of the *lacZ* gene fragment was carried out using the Qiaquick Gel Extraction Kit (Qiagen, Hilden, Germany). The purified *lacZ* fragment was then ligated with the *Eag*I and *Mlu*I digested vector pMSE3 and *E. coli* DH10B was transformed with the resulting recombinant plasmid, yielding plasmid pMSE3 AD1. Three PCRs were necessary to amplify the *acoA*-promoter with enhancer sequence, secretion signal and MCS. The first PCR reaction was carried out with oligonucleotides TB78A/TB78B using genomic DNA from *B. subtilis* 6051 as the template. In two subsequent PCRs with oligonucleotides TB79A/TB79B and TB80A/TB80B, respectively, the amplicon was extended with the downstream box (DB) sequence, the *csn*-encoded signal peptide as well as the *Xho*I and *Bpl*I restriction sites. After digestion with *Xho*I and *Eag*I, the PCR product was gel-purified and ligated with the *Xho*I and *Eag*I restricted vector pMSE3 AD1. *E. coli* DH10B was transformed with the resulting recombinant plasmid yielding the first pMSE3 based toolbox vector, namely pMSE3 P_{acoA} TB1. The *acoA*-promoter without DB enhancer sequence was amplified with oligonucleotides TB83/TB84 using pMSE3 P_{acoA} TB1 as the template. After PCR product digestion with *Xho*I and *Nde*I and subsequent purification, the promoter fragment was ligated with the *Xho*I and *Nde*I digested vector pMSE3 P_{acoA} TB1, thereby replacing the P_{acoA}-DB promoter with the P_{acoA}

promoter without enhancer sequence. After transformation in *E. coli* DH10B, the resulting plasmid was designated pMSE3 P_{acoA} TB2.

The promoter of the *aprE gene* was amplified in a stepwise manner. The first PCR was carried out with oligonucleotides TB85/TB86 using genomic DNA of *B. subtilis* 6051 as the template. The PCR product was gel-purified and served as template for the second PCR with oligonucleotides TB85/TB87, in which the downstream box sequence and the restriction sites were fused to the promoter fragment. After digestion with *Xho*I and *Nde*I, the PCR product was purified and ligated to the *Xho*I and *Nde*I cut vector pMSE3 P_{acoA} TB1, thereby replacing the *acoA* fragment with the *aprE* promoter. *E. coli* DH10B was transformed with the recombinant plasmid, yielding pMSE3 P_{aprE} TB1. For integration of the *aprE* promoter without DB sequence, a PCR with oligonucleotides TB85/TB88 was carried out in which pMSE3 P_{aprE} TB1 served as the template. The PCR product was digested with *Xho*I and *Nde*I and integrated into the *Xho*I and *Nde*I sites of pMSE P_{acoA} TB1. After transformation in *E. coli* DH10B, the resulting plasmid was designated pMSE3 P_{aprE} TB2. In order to transfer both P_{acoA} and P_{aprE} based expression cassettes into the medium copy vector pBE-S and the low copy vector pHB201, the respective pMSE3 vector backbones served as templates for PCR amplification. For construction of the pBE-S vectors, both P_{acoA} regulated expression cassettes were amplified with oligonucleotides TB89/TB95. After digestion of the amplicons with *Spe*I and *Sal*I and subsequent purification, PCR products were ligated into the *Spe*I and *Sal*I sites of pBE-S. *E. coli* DH10B was transformed with the recombinant plasmids yielding pBE-S P_{acoA} TB1 and pBE-S P_{acoA} TB2 respectively. The P_{aprE} regulated expression cassettes were amplified with oligonucleotides TB93/TB95 and were afterwards treated the same way as the corresponding P_{acoA} regulated counterparts. The resulting recombinant plasmids were designated pBE-S P_{aprE} TB1 and pBE-S P_{aprE} TB2. For integration of the expression cassettes into pHB201, the P_{acoA} constructs were amplified with oligonucleotides TB91/TB92 and oligonucleotides TB94/TB92 were used for amplification of the P_{aprE} fusions. All four amplicons were digested with *Hind*III and *Pvu*I, purified and ligated into the *Hind*III and *Pvu*I sites of pHB201. *E. coli* DH10B was transformed with the recombinant plasmids, yielding pHB201 P_{acoA} TB1, pHB201 P_{acoA} TB2, pHB201 P_{aprE} TB1 and pHB201 P_{aprE} TB2. Sequence identity of all 12 vectors with *csn* secretion signal was verified by sequencing (Eurofins Genomics, Ebersberg, Germany).

Construction of toolbox vectors with lipA signal peptide

Based on the vector pMSE3 AD1, which contains the *lacZ* gene fragment of *Pseudoalteromonas haloplanktis* the construction of the vector set with the *lipA* encoded signal peptide was carried out in analogy to the *csn* SP containing vectors, which also served as the DNA templates for PCR reactions. In brief, P_{acoA}-*lipA* fusions with and without *cspB* DB were amplified in two PCR reactions with oligonucleotides TB83/TB103 and TB83/TB104 (with enhancer sequence) and TB83/TB105 plus

TB83/TB104 (without enhancer sequence). After digestion with *Xho*I/*Eag*I and ligation into the *Xho*I/*Eag*I sites of pMSE3 AD1, *E. coli* DH10B was transformed with the recombinant plasmids. The resulting vectors were designated pMSE3 P_{acoA} TB3 and pMSE3 P_{acoA} TB4. The promoter regions of *aprE* were amplified using oligonucleotides TB85/TB87 (with DB) and TB85/TB88 (without DB), digested with *Xho*I/*Nde*I and ligated into the *Xho*I/*Nde*I sites of pMSE3 P_{acoA} TB3. *E. coli* DH10B was transformed with the recombinant plasmids, yielding pMSE3 P_{aprE} TB3 and pMSE3 P_{aprE} TB4. Transfer of the expression cassettes into pBE-S was carried out by amplification of the P_{acoA}-fusions with oligonucleotides TB89/TB95 and subsequent integration into the *Spe*I/*Sal*I sites of pBE-S. The P_{aprE}-fusions were amplified with oligonucleotides TB93/TB95 and treated as described above. The ligation reactions were used for transformation of *E. coli* DH10B, the resulting recombinant plasmids were designated pBE-S P_{acoA} TB3, pBE-S P_{acoA} TB4, pBE-S P_{aprE} TB3 and pBE-S P_{aprE} TB4. Finally, the *lipA* containing expression cassettes were integrated into the low copy vector pHB201. For this purpose, P_{acoA} fusions were amplified with oligonucleotides TB91/TB92 whereas primers TB94/TB92 were used for amplification of the *aprE* promoter fragments. All amplicons were cut with *Hind*III and *Pvu*I, purified and ligated into the *Hind*III/*Pvu*I digested vector pHB201. *E. coli* DH10B was transformed with the recombinant plasmids, yielding pHB201 P_{acoA} TB3, pHB201 P_{acoA} TB4, pHB201 P_{aprE} TB3 and pHB201 P_{aprE} TB4. Sequence identity of all 12 vectors with *lipA* secretion signal was verified by sequencing.

Construction of toolbox vectors with yncM signal peptide

Two PCR reactions were necessary to fuse the *yncM* secretion signal to the *acoA* promoter. The first amplification, using oligonucleotides TB83/TB106 was carried out using pMSE3 P_{acoA} TB1 as the template. The purified PCR product served as template for the second PCR with oligonucleotides TB83/TB107. For the *acoA* promoter without enhancer sequence, amplification was also carried out stepwise, first with oligonucleotides TB83/TB108 and, in a second PCR, with oligonucleotides TB83/TB107. Both amplicons were digested with *Xho*I/*Eag*I and ligated to the *Xho*I/*Eag*I sites of pMSE3 AD1. *E. coli* DH10B was transformed with the ligation reactions. The recombinant vectors were designated pMSE3 P_{acoA} TB5 and pMSE3 P_{acoA} TB6. Amplification of the *aprE* promoter region with and without enhancer sequence was done with oligonucleotides TB85/TB87 (+DB) and TB85/TB88 (-DB), using the pMSE3 P_{aprE} TB1/TB2 templates, respectively. After PCR product digestion with *Xho*I/*Nde*I and subsequent purification, the PCR fragments were ligated to the *Xho*I/*Nde*I digested vector pMSE3 P_{acoA} TB5, thereby replacing the *acoA* promoter with the P_{aprE} fusions. *E. coli* DH10B was transformed with the recombinant plasmids yielding pMSE3 P_{aprE} TB5 and pMSE3 P_{aprE} TB6. Afterwards, all four promoter fragments with *yncM* signal peptide were transferred into the pBE-S and pHB201 backbones. To this purpose, PCRs for pBE-S integrations were carried out with oligonucleotides TB89/TB95 (P_{acoA}) and TB93/TB95 (P_{aprE}) using the respective pMSE3 vectors as templates. PCR products were cut with

*Spe*I/*Sal*I and ligated to the *Spe*I/*Sal*I sites of pBE-S. After transformation of *E. coli* DH10B, the resulting recombinant plasmids were designated pBE-S P_{acoA} TB5, pBE-S P_{acoA} TB6, pBE-S P_{aprE} TB5 and pBE-S P_{aprE} TB6. For construction of the pHB201 vectors, amplification of the different promoter fusions was carried out with TB 91/TB92 (P_{acoA}) and TB94/TB92 (P_{aprE}). After PCR product digestion with *Hind*III/*Pvu*I and subsequent purification, all fragments were ligated into the *Hind*III/*Pvu*I sites of pHB201. *E. coli* DH10B was transformed with the recombinant plasmids, yielding pHB201 P_{acoA} TB5, pHB201 P_{acoA} TB6, pHB201 P_{aprE} TB5 and pHB201 P_{aprE} TB6. Sequence identity of all 12 vectors with *yncM* secretion signal was verified by sequencing.

Construction of toolbox-vectors with ylqB signal peptide

Similar to the *csn*, *lipA* and *yncM* secretion signals, also the *ylqB* signal peptide was fused to the promoter regions of *acoA* and *aprE*. Both fragments, P_{acoA} with and without enhancer sequence comprised two PCR reactions, in which the product of the first PCR served as template for the second amplification. The P_{acoA} fusion with enhancer sequence was amplified successively with oligonucleotides TB83/TB109 and TB83/TB110, whereas oligonucleotides TB83/TB111 and TB83/TB110 were used for the *acoA* promoter fragment without enhancer sequence. Both PCR products were cut with *Xho*I/*Eag*I and the purified fragments were ligated into the *Xho*I/*Eag*I sites of pMSE AD1. *E. coli* DH10B was transformed with the recombinant plasmids yielding pMSE3 P_{acoA} TB7 and pMSE3 P_{acoA} TB8. Amplification of the *aprE* promoter with DB sequence was carried out with oligonucleotides TB85/TB87 and with primers TB85/TB88 for the *aprE* promoter region without enhancer sequence. After PCR product digestion with *Xho*I/*Nde*I and purification, both fragments were ligated into the *Xho*I/*Nde*I sites of pMSE3 P_{acoA} TB7, thereby replacing the *acoA* promoter with the *aprE* promoter variants. After transformation of *E. coli* DH10B, the resulting recombinant plasmids were designated pMSE3 P_{aprE} TB7 and pMSE3 P_{aprE} TB8. The transfer of the *ylqB* fusions into pBE-S and pHB201 was carried out with the same oligonucleotides and restriction enzymes as described before. *E. coli* DH10B was transformed with the recombinant plasmids, yielding pBE-S P_{acoA} TB7, pBE-S P_{acoA} TB8, pBE-S P_{aprE} TB7, pBE-S P_{aprE} TB8, pHB201 P_{acoA} TB7, pHB201 P_{acoA} TB8, pHB201 P_{aprE} TB7 and pHB201 P_{aprE} TB8. Sequence identity of all 12 vectors with *ylqB* secretion signal was verified by sequencing.

Construction of toolbox vectors for intracellular gene expression

A final set of vectors was also designed for intracellular expression. To this end, the *acoA* promoter with enhancer sequence was amplified with oligonucleotides TB83/TB96 using pMSE3 P_{acoA} TB1 as the template. The P_{acoA} fragment without enhancer sequence was amplified from pMSE3 P_{acoA} TB2 DNA with oligonucleotides TB83/TB97. PCR reactions for the P_{acoA} fragment with and without enhancer sequence were carried out with oligonucleotides TB85/TB96 and TB85/TB98, respectively. All four

promoter fusions were digested with *Xho*I/*Eag*I followed by subsequent PCR product purification. After ligation into the *Xho*I/*Eag*I sites of pMSE3 AD1, *E. coli* DH10B was transformed with the recombinant plasmids, yielding pMSE3 P_{acoA} TB9, pMSE3 P_{acoA} TB10, pMSE3 P_{aprE} TB9 and pMSE3 P_{aprE} TB10. As described for the signal peptide containing vectors, the promoter fusions for intracellular expression were transferred into the backbones of pBE-S via *Sal*I/*Spe*I and pHB201 via *Hind*III/*Pvu*I using the same oligonucleotides (TB89/TB95 and TB93/TB95 for pBE-S; TB91/92 and TB94/TB92 for pHB201). After transformation of *E. coli* DH10B, the recombinant plasmids were designated pBE-S P_{acoA} TB9, pBE-S P_{acoA} TB10, pBE-S P_{aprE} TB9, pBE-S P_{aprE} TB10, pHB201 P_{acoA} TB9, pHB201 P_{acoA} TB10, pHB201 P_{aprE} TB9 and pHB201 P_{aprE} TB10. Sequence identity of all 12 vectors for intracellular expression was verified by sequencing.

Construction of toolbox vectors for sox expression

In order to verify and compare Sox production with our toolbox, a set of 12 vectors was designed for expression of the *sox* gene (Table S3). For this purpose, the *sox* coding sequence was integrated into all toolbox vectors which contain the *csn* signal peptide. Amplification of the *sox* nucleotide sequence was carried out with oligonucleotides TB117 and TB100 using pSox as the template. The PCR product was digested with *Bpl*I and *Kpn*I and subsequently gel-purified. After ligation into the *Bpl*I and *Kpn*I sites of pMSE3 P_{acoA} TB1, pMSE3 P_{acoA} TB2, pMSE3 P_{aprE} TB1 and P_{aprE} TB2, *E. coli* DH10B was transformed with the recombinant plasmids, yielding pMSE3 P_{acoA} S1, pMSE3 P_{acoA} S2, pMSE3 P_{aprE} S3 and pMSE3 P_{aprE} S4. All four *sox* expression cassettes were transferred into the backbones of pBE-S via *Sal*I/*Spe*I and pHB201 via *Hind*III/*Pvu*I using oligonucleotides TB89/TB95 and TB93/TB95 for pBE-S; TB91/92 and TB94/TB92 for pHB201. After transformation of *E. coli* DH10B, the recombinant plasmids were designated pBE-S P_{acoA} S5, pBE-S P_{acoA} S6, pBE-S P_{aprE} S7, pBE-S P_{aprE} S8, pHB201 P_{acoA} S9, pHB201 P_{acoA} S10, pHB201 P_{aprE} S11 and pHB201 P_{aprE} S12. Sequence identity of all 12 vectors for *sox* expression was verified by sequencing.

Construction of toolbox vectors for IL expression

In addition to Sox, IL production was also verified with the toolbox setup. For this purpose, the set of 12 IL expression vectors was constructed on the basis of all toolbox plasmids which contain the LipA signal peptide (Table S3). The nucleotide sequence of the IL 1B gene (Genbank Accession number MZ783065) was synthesized and provided by GenScript Biotech (Leiden, Netherlands) and the synthetic IL gene was codon-optimized for expression in *B. subtilis* using the GenSmart™ Codon Optimization tool (GenScript). The algorithm utilizes a matrix for the most frequently occurring codons in *B. subtilis*. The construct was assembled from synthetic oligonucleotides and provided in the backbone of the pUC19 vector (pUC19-IL1B-co). Amplification of the IL nucleotide sequence was

carried out with oligonucleotides TB112 and TB113 using pUC19-IL1B-co as the template. The PCR product was digested with *Bpl*I and *Kpn*I and subsequently gel-purified. After ligation into the *Bpl*I and *Kpn*I sites of pMSE3 P_{acoA} TB3, pMSE3 P_{acoA} TB4, pMSE3 P_{aprE} TB3 and P_{aprE} TB4, *E. coli* DH10B was transformed with the recombinant plasmids, yielding pMSE3 P_{acoA} IL1, pMSE3 P_{acoA} IL2, pMSE3 P_{aprE} IL3 and pMSE3 P_{aprE} IL4. All four IL expression cassettes were transferred into the backbones of pBE-S via *Sal*I/*Spe*I and pHB201 via *Hind*III/*Pvu*I using oligonucleotides TB89/TB95 and TB93/TB95 for pBE-S; TB91/92 and TB94/TB92 for pHB201. After transformation of *E. coli* DH10B, the recombinant plasmids were designated pBE-S P_{acoA} IL5, pBE-S P_{acoA} IL6, pBE-S P_{aprE} IL7, pBE-S P_{aprE} IL8, pHB201 P_{acoA} IL9, pHB201 P_{acoA} IL10, pHB201 P_{aprE} IL11 and pHB201 P_{aprE} IL12. Sequence identity of all 12 IL expression vectors was verified by sequencing.

Protein purification

Sox purifications for activity measurements were carried out by small-scale purification applying Ni-NTA Spin Columns (Qiagen) as proposed by the manufacturer, but with a starting volume of 2.4 mL supernatant. Large-scale protein purification was also performed with Ni-NTA affinity chromatography according to the respective QIAexpressionist protocol with a low-pressure Econo chromatography system (Bio-Rad). In brief, the culture supernatant was adjusted to pH 8.0, centrifuged and loaded onto the Ni-NTA column. After consecutive washing with lysis buffer (50 mM NaH_2PO_4, 300 mM NaCl, 10 mM imidazole, pH 8.0) and wash buffer (50 mM NaH_2PO_4, 300 mM NaCl, 20 mM imidazole, pH 8.0), Sox was eluted with elution buffer (50 mM NaH_2PO_4, 300 mM NaCl, 250 mM imidazole, pH 8.0) and dialyzed against 50 mM NaH_2PO_4, 300 mM NaCl, pH 8.0.

Sox assay

In order to avoid interferences with media components, Sox activity obtained from shaking flask experiments and initial batch fermentations was assayed with precipitated culture supernatants. Precipitation was carried out with 40% ammonium sulfate on ice for 1 h followed by centrifugation and subsequent resuspension of the pellet in PBS, pH 7.4. In case of comparative batch and fed-batch fermentation setups, Sox was subjected to at-line small-scale protein purification as describe above. For Sox activity screening, HisSorb Plates (Qiagen) were applied for micro-scale purification as recommended by the manufacturer.

Sox was assayed by two different methods: Either by measuring the disappearance of thiol groups or by measuring hydrogen peroxide formation with a peroxidase-coupled assay (Janolino and Swaisgood 1975; Raje et al. 2002; Faccio et al. 2010). When applying the first method, the concentration of thiol groups was determined with 5,5'-dithiobis-(2-nitrobenzoic acid) (DTNB, Ellman's reagent) as described by Ellman prior to each measurement (Ellman 1958). The reaction mixture contained dithiothreitol

(DTT) as substrate and the purified enzyme in PBS, pH 7.4 supplemented with 3 mM EDTA. Here, DTT was applied at a concentration of 0.3 mM to yield an initial absorption of approximately 0.8 at 412 nm. After 15 min incubation at 30 °C, the reaction was stopped by adding Ellman's reagent. The absorbance at 412 nm was determined and compared to a negative control, which was obtained by adding the enzyme after stopping the reaction. The difference in absorption between both values was then used to calculate volumetric enzyme activity applying a molar extinction coefficient ε_{TNB} of 13,600 M^{-1} cm^{-1}. The assay was applied in cuvettes for the analysis of enzyme activity during fermentation experiments, but also downscaled to micro-scale for application in microtiter plates for enzyme characterization as described below. 20 µL enzyme were incubated with 80 µL substrate mixture containing 0.02 mM DTT in PBS, pH 7.4 supplemented with 3 mM EDTA. The reaction was stopped after 0, 5, 10 and 15 min by addition of 100 µL Ellman's reagent applying a Freedom Evo® screening robot (Tecan) equipped with a Safire2 plate reader. In this case, enzyme activity was determined from the obtained slope of this semi-continuous assay. 1 unit of enzyme activity was defined as the amount of enzyme oxidizing 1 µmol of thiol groups per minute.

For Sox activity screening in micro-scale, a continuous Sox assay was applied as described previously (Faccio et al. 2010). The assay was performed with 10 µL enzyme and 150 µL reaction mixture consisting of 1 mM homovanillic acid (HVA), 1.4 µM horseradish peroxidase (HRP) and 0.3 mM DTT in 50 mM potassium phosphate, pH 7.5 supplemented with 3 mM EDTA. The formation of fluorescent HVA dimer was continually monitored applying an excitation wavelength λ_{ex} of 320 nm and an emission wavelength λ_{em} of 420 nm in black microtiter plates. Here, enzyme activity was determined from the obtained slope based on a calibration curve obtained with different hydrogen peroxide concentrations. 1 unit of enzyme activity was defined as the amount of enzyme producing 1 µmol hydrogen peroxide per minute.

Cultivation conditions and media of the fermentation experiments

Pre-cultures for fermentation experiments were grown in 500 mL-shaking flasks without baffles containing 50 mL adjusted Neubauer Minimal Medium (NBMM) consisting of a synthetic basal medium with 1.5 g/L Na_2SO_4, 2,47 g/L $(NH_4)_2SO_4$, 0.5 g/L NH_4Cl, 1 g/L $(NH_4)_2$-citrate, 10.625 g/L K_2HPO_4, 5.38 g/L NaH_2PO_4 * 2 H_2O as well as 15 g/L yeast extract and 3.2 g/L peptone. The medium was adjusted to pH 7.0, sterilized by autoclaving and supplemented with a trace element solution as proposed by Neubauer et al. (1995) and final concentrations of 2 mM $MgSO_4$, 68 µM $CaCl_2$ * 2 H_2O, 2 g/L or 10 g/L glucose, 0.05 % or 0.5 % (w/v) acetoin and 30 µg/mL Kanamycin (Neubauer et al. 1995a; Neubauer et al. 1995b). Pre-cultures were grown for 24 h at 37 °C with shaking at 160 rpm. Growth experiments in small scale were performed accordingly. Fermentations were carried out with NBMM either in a parallel fermentation setup applying foil fermenters with 1.5 L culture volume or in a steel stirred tank

reactor with a working volume of 10 L (Bioengineering AG). Controlled parameters during the fermentations were pH (adjusted to pH 7.0), aeration (60 L air/h, corresponding to 0.75 vvm when applying a 1.5 L working volume), impeller speed (set to 800 rpm at fermentation start, then variable according to dissolved oxygen level) and a cultivation temperature of 37 °C. Anti-foam agent Desmophen® was manually applied when appropriate. Batch and fed-batch fermentation strategies with varying concentrations of glucose, peptone and yeast extract as well as the inductor acetoin were tested as indicated for each experiment.

Results

Identification of suitable secretion signals

In order to determine suitable secretion signals for Sox secretory overproduction, we used a secretion expression system from Takara Clontech ("*B. subtilis* Secretory Protein Expression System"), which allows the fusion of 173 Sec-dependent signal peptides of *B. subtilis* to the gene of interest. A total of 288 *Bacillus* clones was screened for Sox production by fluorescent based activity assay. An increase in relative fluorescence units (RFU) indicated an activity of the Sox enzyme in this prescreening (Fig. S2). Thus, 12 clones with positive ΔRFU values were identified and compared to one clone with unaltered fluorescence (ΔRFU=0) and a negative control (ΔRFU<0) by SDS Page analysis (Fig. S3a). Prior to this, the secretion signals of all selected Sox clones were identified by sequencing.

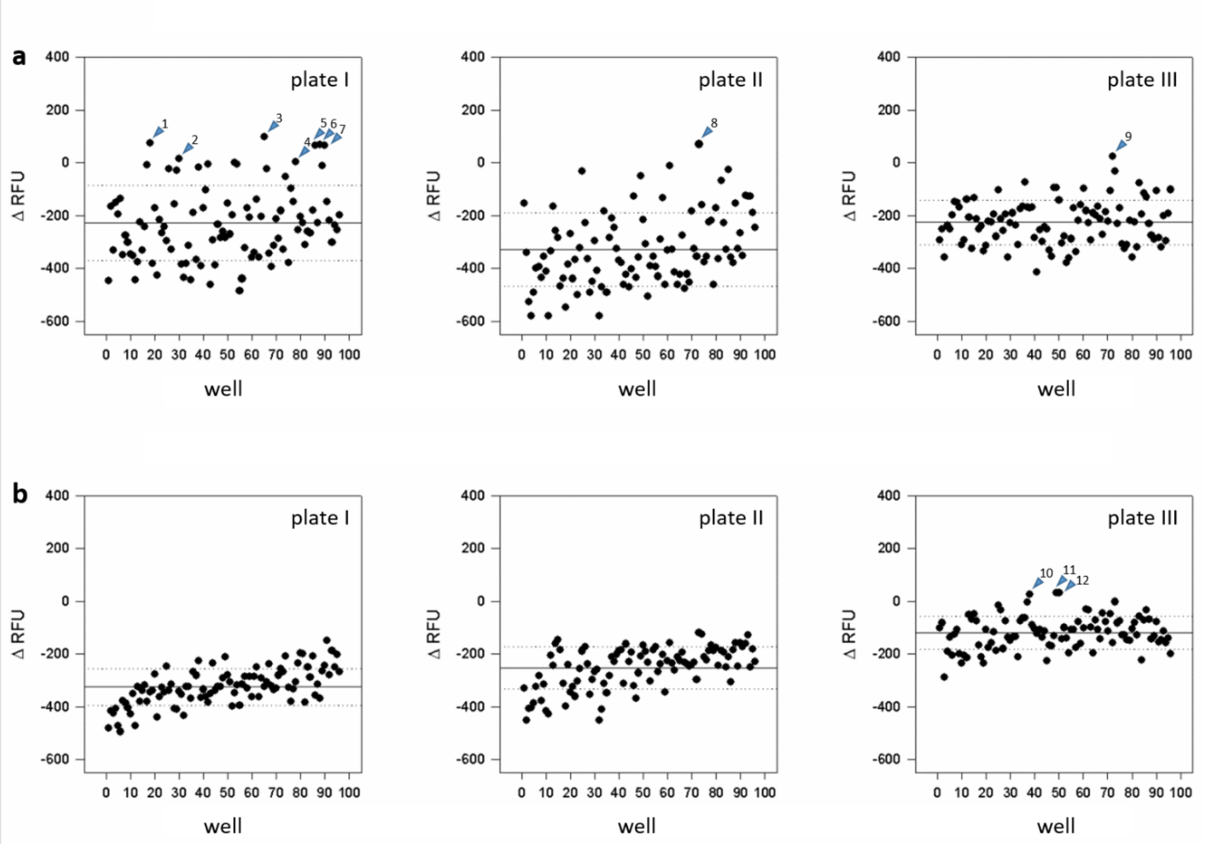

Figure S2 Activity-based prescreening of 288 Sox clones for efficient secretion. ΔRFU values of three 96 well plates after cultivation of Sox clones in EnpressoB medium after 24 h (**a**) and 48 h (**b**). Selected signal peptide constructs, which showed increased secreted levels of the target protein, are marked as follows: 1-YwmC-Sox, 2-PelB-Sox, 3-YhzA-Sox, 4-NucB-Sox, 5-YfkD-Sox, 6-YweA-Sox, 7-YlqB-Sox, 8-Csn-Sox, 9-YbdN-Sox, 10-SelB-Sox, 11-YxaK-Sox, 12-YhjA-Sox.

Using sulfhydryl oxidase as a model enzyme, we were able to identify >10 signal peptide sequences that mediated Sox secretion (Fig. S3a). Among them, the signal peptides of Csn, NucB, YweA and YlqB apparently mediated highest Sox yields under P_{aprE}-controlled expression in the pBE-S vector backbone. For this reason, these four signal peptide sequences were also integrated in our pMSE3-based expression vector with the P_{acoA} expression system in order to compare secretion efficiencies/protein yields of these newly identifies signal peptides with the secretion signal of AmyE in pSox (Table 2). During cultivation of the *sox* expression strains in EnpressoB at 37 °C, sampling was carried out 24 h and 48 h after the boost and the Sox-containing supernatant was purified using the ProCatch His Resin (Miltenyi Biotech, Teterow, Germany). Protein amounts were visualized and quantified (Fig. S3b). The results of these experiments were consistent and revealed strong distinct protein bands of the secreted Sox protein with the four signal peptides of YweA, NucB, YlqB and Csn, with the latter one contributing to the highest protein amounts. The amount of Sox protein secreted by the control signal peptide of AmyE in these experiments was significantly lower.

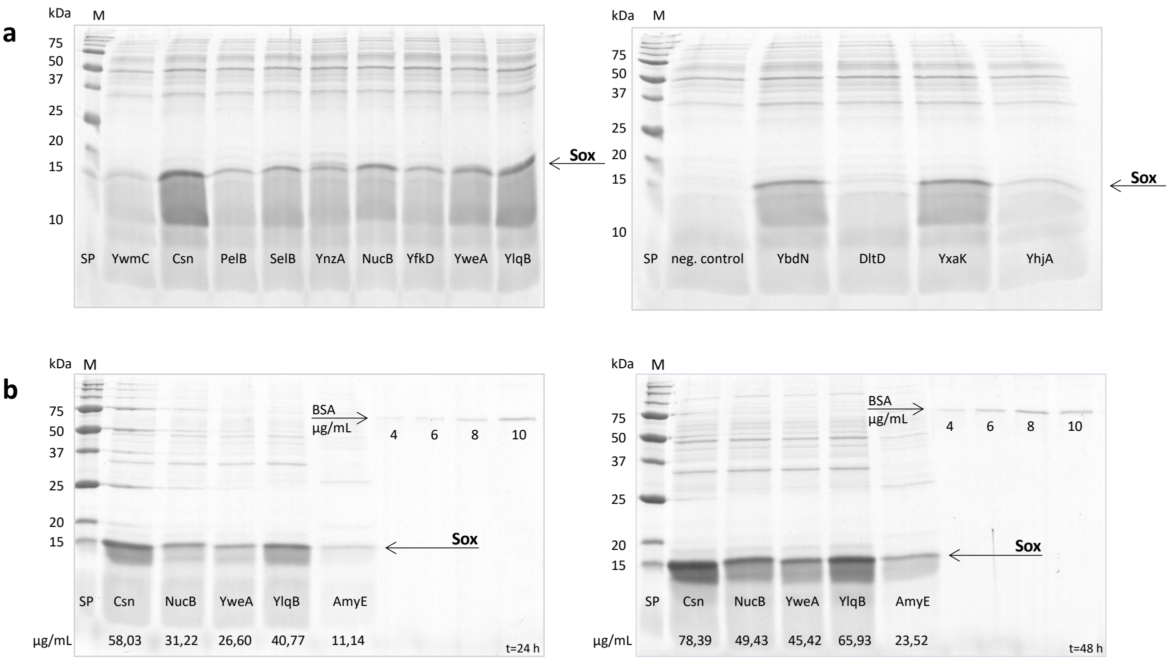

Figure S3 (**a**) Screening of suitable signal peptide sequences for efficient Sox secretion. Exemplarily, clones are shown which revealed highest Sox production and secretion activity after 48 h in *B. subtilis* LS8P-D Δ*amyE* in comparison to a negative control and one clone with unaltered fluorescence (*dltD*-SP). The respective secretion signal (SP) is indicated below each lane. (**b**) Sox protein amounts (µg/mL) after purification of 1 mL supernatant after 24 h (left) and 48 h (right). In order to quantify protein amounts, a BSA standard with known concentrations (4-10 µg/mL) was additionally loaded on the gel.

Verification of the toolbox for the production of interleukin

For human interleukin-1β (IL), the second model protein for the evaluation of our toolbox, the experimental setup was equal to the *sox* expression procedures. To gain an overview about the influence of different toolbox modules on the overproduction of IL in *B. subtilis*, similar to the *sox* constructs a set of 12 expression strains (see Table 3) for this target protein was analyzed under small-scale conditions in EnpressoB medium (Fig. S4a-c). Based on the calibration curve derived from the BSA standard, signal intensities of all interleukin samples from the culture supernatant were converted into protein amounts (Fig. S4d-f). 24 h after the boost, the overall highest interleukin protein amounts with average values of 2-2.5 µg/mL in the culture supernatant were detected for the IL9-12 strains with the low-copy vector pHB201 (Fig. S4f). Similar values were calculated for the pMSE3-based high-copy vectors regulated by the inducible P_{acoA} promoter (strains IL1, IL2), whereas the P_{aprE} regulated expression strains IL3 and IL4 revealed lower IL amounts (Fig. S4d). For pBE-S based interleukin production, highest amounts of the target protein (2 µg/mL) were calculated for the IL6 strain. After prolonged growth at 48 h, a significant increase in IL amounts (approx. 3 µg/mL) was detected for the IL2 construct and to a lesser extent for the IL9-11 strains (Fig. S4d). Thus, highest IL production was reached by the high copy vector also with the acetoin controlled P_{acoA} promoter.

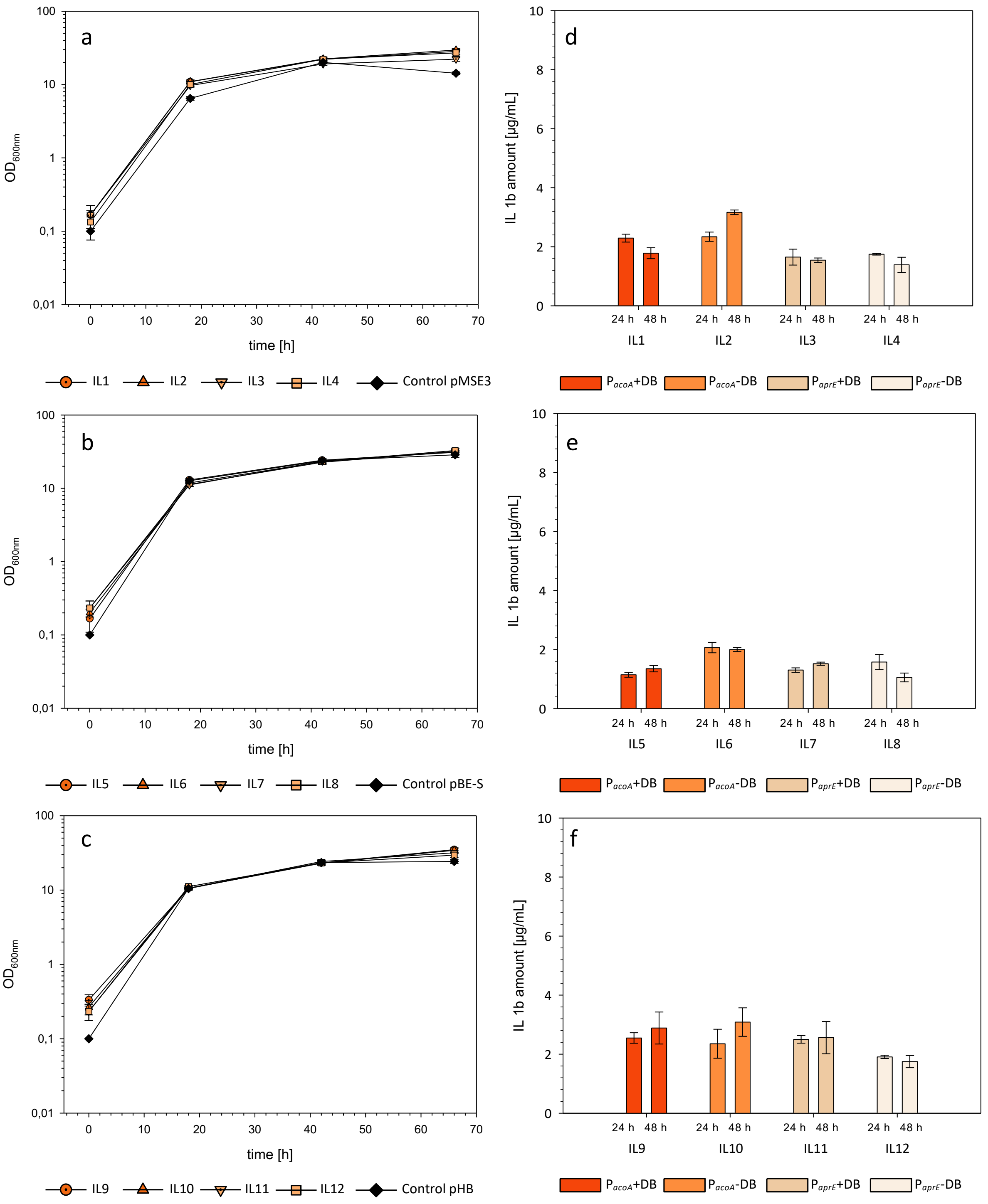

Figure S4 Verification of the toolbox strains IL1-IL12 (see also Table 3) for the overexpression of the human growth factor interleukin-1β. (**a-c**) Growth curves of *B. subtilis* JK139 IL1-IL12 in comparison to the negative control carrying the "empty vector" without expression cassette. Cultivations were performed in biological triplicates; standard deviations are presented as error bars. (**d-f**) Quantitative analyses of the secretory overproduction of IL. Average protein amounts (normalized by OD) for the IL strains IL1-IL12 in the culture supernatant 24 h and 48 h after the boost in EnpressoB medium. Protein concentrations are given as average values from three independent experiments. Standard deviations are presented as error bars. For comparison of protein yields, the target protein was quantified. "Empty vector" - strains without expression cassette served as negative controls.

Development of a fermentation strategy for Sox and IL production

Identification of cultivation parameters in shake flasks

Analysis of essential cultivation parameters was the first step towards Sox production at larger scales. To analyze the influence of temperature on Sox production, *B. subtilis* WB600 pSox was grown in shaking flasks at 30 °C and 37 °C (Fig. S5a). Growth was determined by measuring optical density at 600 nm (OD_{600nm}). Expression of the *sox* gene was induced by initial addition of 0.5 % (w/v) acetoin. Sox production was visualized by SDS-PAGE analysis and by determining Sox activity. It is shown that both temperatures resulted in similar cell densities of about 7.8 at 30 °C and 8.0 at 37 °C after 12 h and final Sox activities of 15.5 U/L (30 °C) and 13.7 U/L (37 °C) after 24 h, respectively (Fig. S5a). However, at 37 °C the cells grew slightly faster and were also found to start producing the target enzyme earlier. This was additionally shown by SDS-PAGE analysis (Fig. S5b).

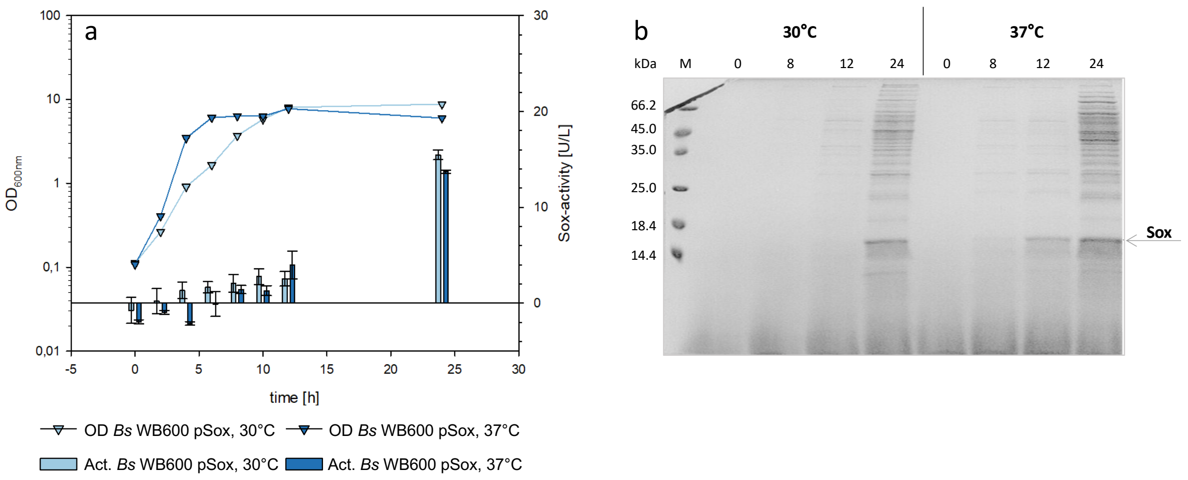

Figure S5 Influence of temperature on Sox production in *B. subtilis* WB600 pSox. (**a**) Cells were grown in shaking flasks at 30 °C (light blue) and 37 °C (dark blue) in NBMM containing 2 g/L glucose and 0.5 % (w/v) acetoin. Cell growth was monitored by the determination of the OD at 600 nm (triangles). Sox activity was determined in triplicates using precipitated culture supernatants (bars). (**b**) Influence of temperature on Sox production: SDS-PAGE analysis of untreated culture supernatant for excreted target protein.

Upscaling from shake flask to fermenter

A scale-up to a parallel 1.5 L batch fermentation setup was performed at 37 °C and simultaneously used to test the influence of a higher glucose concentration on growth and productivity of *B. subtilis* WB600 pSox (Fig. S6a). Growth curves for cultivations with 2 g/L or 10 g/L glucose were determined as described above. Sox production was monitored by activity measurements and SDS-PAGE analysis. In comparison to higher glucose concentrations, a glucose concentration of 2 g/L resulted in increased activities of the target enzyme with 16.5 U/L compared to 12.2 U/L obtained from the batch

fermentation with 10 g/L glucose after 12 h. A lower glucose concentration in the medium also resulted in higher optical densities with maximum values of 24.6 after 8 h compared to 18.9 after 6 h with 10 g/L glucose. However, both cultivation conditions were found to provoke high autolysis rates of *B. subtilis* WB600 pSox. At a higher glucose concentration, the production strain showed an increased growth rate and an earlier start of the stationary phase in the applied batch fermentation setup (Fig. S6a). SDS-PAGE analysis of the secreted proteome indicated a similar start of Sox production regardless of the applied glucose concentration after 8 h of the fermentation process (Fig. S6b).

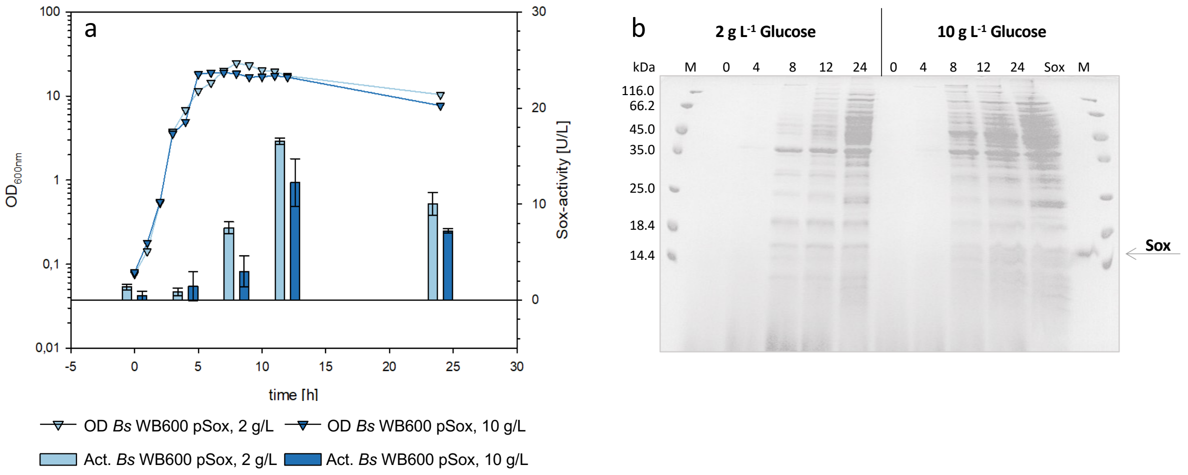

Figure S6 Upscaling and influence of the glucose level on Sox production in *B. subtilis* WB600 pSox. (**a**) The cells were grown again in a batch fermentation at 1.5 L scale at 37 °C in NBMM containing 2-fold concentrated complex components yeast extract and peptone (30 and 6.4 g/L, respectively), 0.5 % (w/v) acetoin and either 2 or 10 g/L glucose. Cell growth was monitored by the determination of the OD at 600 nm (triangles). Sox activity was determined in triplicates using precipitated culture supernatants (bars). (**b**) SDS-PAGE analysis of untreated culture supernatant for excreted target protein.

Overproduction of the human growth factor interleukin-1β

We used the acetoin inducible promoter and the novel *B. subtilis* expression strain JK139 to test the best performing toolbox setup IL2 (see Fig. S4d) with the new fed-batch fermentation strategy for interleukin overproduction. Fig. S7 illustrates the calculated protein amounts of IL in the culture supernatant after upscaling. IL production increased over the whole fermentation time with maximum values of approximately 3 µg/mL after 48 h. In contrast to Sox production, comparable IL protein amounts were calculated for small- and large-scale cultivations. In comparison to the overproduction of other interleukin types in *Bacillus* host vector systems the yield of the IL-1β target protein is relatively low (Takimura et al. 1997; Shiga et al. 2000; Westers et al. 2006). However, it is worth mentioning that this interleukin type has never been produced in *B. subtilis* so far. Thus, a direct

comparison with other expression systems is difficult. Our study reveals that it is able to overproduce this human protein with the established *B. subtilis* expression and secretion platform.

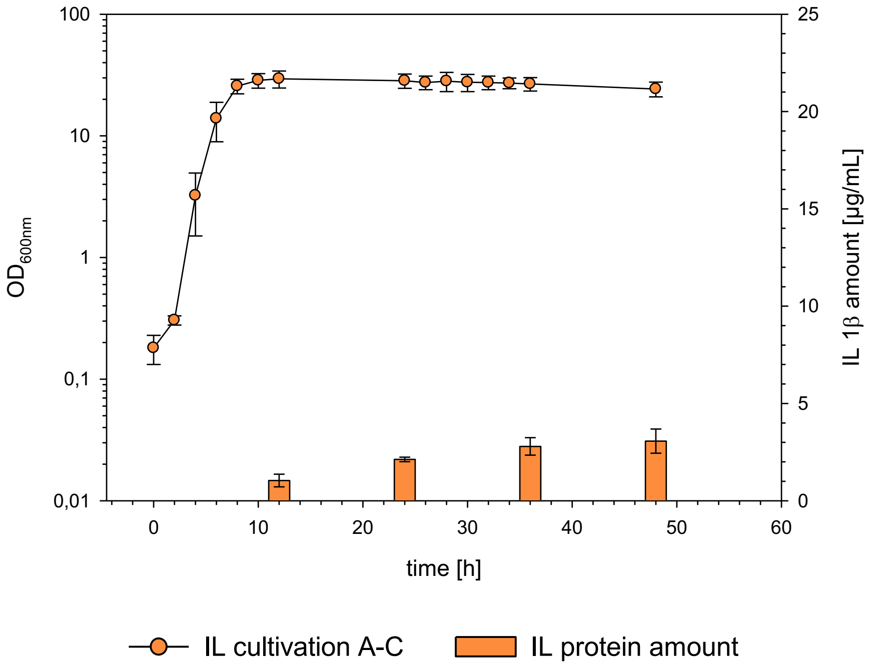

Figure S7 Verification of the best performing IL2 toolbox setup in *B. subtilis* JK139 for the overexpression of the human growth factor interleukin IL-1β. The growth curve reflects three individual 1.5 L fed-batch fermentation experiments A-C. Shown are average protein amounts (normalized by OD) from the culture supernatant of the IL2 expression strains from three independent experiments. Standard deviations are presented as error bars.

References

Ellman GL (1958) A colorimetric method for determining low concentrations of mercaptans. Archives of Biochemistry and Biophysics 74:443–450. doi: 10.1016/0003-9861(58)90014-6

Faccio G, Kruus K, Buchert J, Saloheimo M (2010) Secreted fungal sulfhydryl oxidases: sequence analysis and characterisation of a representative flavin-dependent enzyme from Aspergillus oryzae. BMC Biochem 11:31. doi: 10.1186/1471-2091-11-31

Janolino VG, Swaisgood HE (1975) Isolation and characterization of sulfhydryl oxidase from bovine milk. J Biol Chem 250:2532–2538

Kabisch J, Thürmer A, Hübel T, Popper L, Daniel R, Schweder T (2013) Characterization and optimization of Bacillus subtilis ATCC 6051 as an expression host. J Biotechnol 163:97–104. doi: 10.1016/j.jbiotec.2012.06.034

Neubauer P, Åhman M, Törnkvist M, Larsson G, Enfors S-O (1995a) Response of guanosine tetraphosphate to glucose fluctuations in fed-batch cultivations of Escherichia coli. J Biotechnol 43:195–204. doi: 10.1016/0168-1656(95)00130-1

Neubauer P, Häggström L, Enfors SO (1995b) Influence of substrate oscillations on acetate formation and growth yield in Escherichia coli glucose limited fed-batch cultivations. Biotechnol Bioeng 47:139–146. doi: 10.1002/bit.260470204

Raje S, Glynn NM, Thorpe C (2002) A continuous fluorescence assay for sulfhydryl oxidase. Analytical Biochemistry 307:266–272. doi: 10.1016/S0003-2697(02)00050-7

Shiga Y, Maki M, Ohta T, Tokishita S, Okamoto A, Tsukagoshi N, Udaka S, Konishi A, Kodama Y, Ejima D, Matsui H, Yamagata H (2000) Efficient production of N-terminally truncated biologically active human interleukin-6 by Bacillus brevis. Biosci Biotechnol Biochem 64:665–669. doi: 10.1271/bbb.64.665

Takimura Y, Kato M, Ohta T, Yamagata H, Udaka S (1997) Secretion of human interleukin-2 in biologically active form by Bacillus brevis directly into culture medium. Biosci Biotechnol Biochem 61:1858–1861. doi: 10.1271/bbb.61.1858

Welsch N, Homuth G, Schweder T (2012) Suitability of different β-galactosidases as reporter enzymes in Bacillus subtilis. Appl Microbiol Biotechnol 93:381–392. doi: 10.1007/s00253-011-3645-0

Westers L, Dijkstra DS, Westers H, van Dijl JM, Quax WJ (2006) Secretion of functional human interleukin-3 from Bacillus subtilis. J Biotechnol 123:211–224. doi: 10.1016/j.jbiotec.2005.11.007

Zobel S, Kumpfmüller J, Süssmuth RD, Schweder T (2015) Bacillus subtilis as heterologous host for the secretory production of the non-ribosomal cyclodepsipeptide enniatin. Appl Microbiol Biotechnol 99:681–691. doi: 10.1007/s00253-014-6199-0

Supplemental Tables

Supplemental Table S1: List and sequences of primers used in this study.

Primer	Sequence 5´→ 3´
SO17	ACGTACGGATCCGCATTATCACCATCGGCCTG
SO18	ACGTACTCTAGACGCTTTTGGATTCGCTTGAC
SO21	ACGTACGGATCCGATTGCAACCGGCTTTATCG
SO22	ACGTACTCTAGAGTAAATGATCATTACCGGTG
SO29	ACGTACGGATCCGCGGATTACACTGTTGAAGG
SO30	ACGTACTCTAGACCTCTGTTAACGAGATGTG
SO33	ACGTACGCATGCGGACTTAACGAAGCCTTCTTC
SO34	ACGTACTCTAGACTCCTTAAATGGCGTTATATC
SO35	ACGTACGGATCCCGAAAGAGACAAATCTAATC
SO36	ACGTACGGTACCCCGCGGATCTTGCTTCTGTG
SO38	ACGTACGGTACCTAAACGGCACCTCAAACGGTG
SO39	ACGTACGGATCCGGTTGTTTAGCAAAATGCAG
SO40	ACGTACTCTAGACCAAAAACCTTTAAGATTTGC
SO41	ACGTACGCATGCGACGGGTCGTACAATGGCTG
SO42	ACGTACGGTACCAGGAGATTGTCAAGATTTCGCG
SO43	ACGTACGGATCCGAAGGGCAGAGATATTTCGC
SO44	ACGTACTCTAGAGAGCCGATGGCTGAACTGATTG
SO45	TCCGGCATGCTGCCGATCTCTGTATCAACGG
SO46	ACGTACGGTACCGCAGCACATATCCGTATAAC
SO47	ACGTACGGATCCGATAGTATTGTCCTGTGTTTG
SO48	ACGTACTCTAGATATTAGGAAAAGCCTGAGATCCC
SO49	TCCGGCATGCCTGATGTCGATAAACTCTTT
SO50	ACGTACGGTACCCTCCATTTTCTTCTGCTATC
SO51	ACGTACGGATCCTCTCTATTTAGGTATATCATC
SO52	ACGTACTCTAGATAGTAAAAAGAAGCAGGTTCCTCC
SO53	TCCGGCATGCGGGCCACGAAATGGGCCATTATG
SO56	ACGTACGGTACCGATACAAGCAAACGTTTCATAG
SO57	ACGTACGGATCCCCTTGTGTCATTTGGACCATT
SO58	ACGTACTCTAGAGAAAAAGCCCTGCCGATTCGG
SO59	TCCGGCATGCCTGCGCCGCATATTTCAACGGC
TB20	ACGTACCATATGCCTGGCTCAAGAACATACAGG
TB21	ACGTACTCTAGACTCGAGTTCGTCCCAGCCGTCC
TB76	GCCGAATAATTATCAAGTTGCGGTCCAGATTTTTGATGGCAAAACATCACTGTGCGAACCGAAAATTCAGAGCACAAACAACAA ACGTGTTGATG
TB77	AGATGCTGTTGGCTATATTTTGCTTCAAACAGTGACGGATGCGGTGTTCTATCCGGAAACAGCAGGCCATTAATGCAAAACTGG CGGTCGTTAAGTTC
TB78A	GAACTAGGAAGTTAAAAAGATTTCCAAGGAAATAAATACGTCGATCATTGTCAAAGGCCGGGTGATATCCGGTCTTTTTTTTCC ATGCTG
TB78B	CTTTCTACTAGAAACCTTTTTCAGAGTTGAACCATTTTACTTTACCTTCTAACATGAAATTTCCTCCTAATATTTAGGGTTCAC CATTATAAATG
TB79A	CGGGGACGGCGGTATTGGATATGTCAAATATTCAATTGATTTTCTTTCAAAAGCAGCTATTCAGTCAAACGATGCAGAGGAACT AGGAAGTTAAAAAG
TB79B	CAAGTAATGAGATCGCTGCTTTTTTCCAAAAATCTGCTTTTTGCATACTGATTTTCATATGTTTCACCTCCTTTCTACTAGAAA CCTTTTTCAGAG
TB80A	ACGTACCTCGAGACGGTCAGAAAAACGCTTAATGAAAAGGGATATACATACTCAAACGGCACGATAGACTGTATGCAGAAGGAC GGGGACGGCGGTATTG
TB80B	ATTCGGCGGCCGGCGAGGCATGCTCGCGAGCTCCGCAAAAACCGTTTCGCTCATCATCAGGGTAAAAAACATGGTGAAAACAAG TAATGAGATCGCTG
TB81	CTCGCGAGCATGCCTCGCCGGCCGCCGAATAATTATCAAGTTGCGGTCCAG
TB82	ACGTACACGCGTATAGAAAAAGAGCATTTTTTGAAACAAAACTTCAAAAAATGCTCTTTTTGCTTATTTAGGTACCAGATGCTG TTGGCTATATTTTGC
TB83	ACGTACCTCGAGACGGTCAGAAAAACGCTTAATGAAAAGGG
TB85	ACGTACCTCGAGCATCTGATGTCTTTGCTTGGCG
TB86	CCTTTTTCAGAGTTGAACCATTTTACTTTACCTTCTAACATGAAATTTCCTCCTAATGCTTAAGTTCAGAGTAGACTTACTTAA AAGACTATTC

TB87	ACGTACCATATGTTTCACCTCCTTTCTACTAGAAACCTTTTTCAGAGTTGAACC
TB88	ACGTACCATATGTTTCACCTCCTTTTGCTTAAGTTCAGAGTAGACTTACTTAAAAGACTATTC
TB89	ACGTACACTAGTCTCGAGACGGTCAGAAAAACGCTTAATGAAAAGG
TB91	ACGTACAAGCTTCTCGAGACGGTCAGAAAAACGCTTAATGAAAAGG
TB92	ACGTACCGATCGACGCGTATAGAAAAAGAGCATTTTTTGAAACAAAACTTC
TB93	ACGTACACTAGTCTCGAGCATCTGATGTCTTTGCTTGGCGAATGTTC
TB94	ACGTACAAGCTTCTCGAGCATCTGATGTCTTTGCTTGGCGAATGTTC
TB95	ACGTACGTCGACACGCGTATAGAAAAAGAGCATTTTTTGAAACAAAACTTC
TB96	CTTGATAATTATTCGGCGGCCGGCGAGGCATGCTCGCGAGCTCCATATGTTTCACCTCCTTTCTACTAGAAACCTTTTTCAGAG TTGAACC
TB97	CTTGATAATTATTCGGCGGCCGGCGAGGCATGCTCGCGAGCTCCATATGTTTCACCTCCTTTTATTTAGGGTTCACCATTATAA ATGC
TB98	CTTGATAATTATTCGGCGGCCGGCGAGGCATGCTCGCGAGCTCCATATGTTTCACCTCCTTTTGCTTAAGTTCAGAGTAGACTT ACTTAAAAGAC
TB103	CAGACAGCATCAAAATTGTTACAAGTGCAATGATCCTTCTTTTTACAAATTTCATATGTTTCACCTCCTTTCTACTAGAAACCT TTTTCAGAGTTG
TB104	ATTCGGCGGCCGGCGAGGCATGCTCGCGAGCTCAGCGGCTTTTGCTGACGGCTGCAACGCAAACAGCGATGTAACAGACAGCAT CAAAATTG
TB105	CAGACAGCATCAAAATTGTTACAAGTGCAATGATCCTTCTTTTTACAAATTTCATATGTTTCACCTCCTTTTATTTAGGGTTCA CC
TB106	GAACTGCTGTTCCTACTGCACCTGCAATCAATACTTTTTTCACCAAAATTCCCCCTTTTGATAGTGGTTTCGCCATATGTTTCA CCTCCTTTCTACTAG
TB107	TCGGCGGCCGGCGAGGCATGCTCGCGAGCTCAGCGTCTGCCGCGGGTAAACCTGGTATACCTGATGAAAGGGTTCCGAAAAGAA CTGCTGTTCCTACTGC
TB109	CCTATTGTAAACAGGGCTGCCAGACATAACATAAACAATAAACCGATTTTTTTCATATGTTTCACCTCCTTTCTACTAGAAACC TTTTTCAGAGTTG
TB110	ATTCGGCGGCCGGCGAGGCATGCTCGCGAGCTCAGCGTCTGCTTGCTGTGCCGGGAAACCTATTGTAAACAGGGCTGCCAG
TB111	CCTATTGTAAACAGGGCTGCCAGACATAACATAAACAATAAACCGATTTTTTTCATATGTTTCACCTCCTTTTATTTAGGGTTC ACC
TB112	GTTGCAGCCGTCAGCAAAAGCCGCTGAGCTCGCGAGCATGCCTCGCCGGCCGCCGCTGCCCCGGTTAGATCTTTAAATTGCACA CTGCGCGATAGC
TB113	GCTCTTTTTGCTTATTTAGGTACCTTAGTGGTGATGATGGTGATGGGAGGAGACAAACTGCATCGTAAAATCTGTAATATCTTG AC
TB117	GATGATGAGCGAAACGGTTTTTGCGGAGCTCGCGAGCATGCCTCGCCGGCCGTTGCGATGCCTGGCTCAAGAACATACAGGAAG GTTGACCCTCCTGAC

Supplemental Table S2: Overview of all toolbox vectors

Nos.	Plasmid nomenclature	Relevant genetic elements	Nos.	Plasmid nomenclature	Relevant genetic elements
1	pMSE3 P_{acoA} TB1	P_{acoA}, *DB, csn-SP, Ph-lacZ,*T_{bkdB}	31	pBE-S P_{aprE} TB5	P_{aprE}, *DB, yncM -SP, Ph-lacZ,*T_{bkdB}
2	pMSE3 P_{acoA} TB2	P_{acoA}, *csn-SP, Ph-lacZ,*T_{bkdB}	32	pBE-S P_{aprE} TB6	P_{aprE}, *yncM -SP, Ph-lacZ,*T_{bkdB}
3	pMSE3 P_{aprE} TB1	P_{aprE}, *DB, csn-SP, Ph-lacZ,*T_{bkdB}	33	pHB201 P_{acoA} TB5	P_{acoA}, *DB, yncM -SP, Ph-lacZ,*T_{bkdB}
4	pMSE3 P_{aprE} TB2	P_{aprE}, *csn-SP, Ph-lacZ,*T_{bkdB}	34	pHB201 P_{acoA} TB6	P_{acoA}, *yncM -SP, Ph-lacZ,*T_{bkdB}
5	pBE-S P_{acoA} TB1	P_{acoA}, *DB, csn-SP, Ph-lacZ,*T_{bkdB}	35	pHB201 P_{aprE} TB5	P_{aprE}, *DB, yncM -SP, Ph-lacZ,*T_{bkdB}
6	pBE-S P_{acoA} TB2	P_{acoA}, *csn-SP, Ph-lacZ,*T_{bkdB}	36	pHB201 P_{aprE} TB6	P_{aprE}, *yncM -SP, Ph-lacZ,*T_{bkdB}
7	pBE-S P_{aprE} TB1	P_{aprE}, *DB, csn-SP, Ph-lacZ,*T_{bkdB}	37	pMSE3 P_{acoA} TB7	P_{acoA}, *DB, ylqB-SP, Ph-lacZ,*T_{bkdB}
8	pBE-S P_{aprE} TB2	P_{aprE}, *csn-SP, Ph-lacZ,*T_{bkdB}	38	pMSE3 P_{acoA} TB8	P_{acoA}, *ylqB -SP, Ph-lacZ,*T_{bkdB}
9	pHB201 P_{acoA} TB1	P_{acoA}, *DB, csn-SP, Ph-lacZ,*T_{bkdB}	39	pMSE3 P_{aprE} TB7	P_{aprE}, *DB, ylqB -SP, Ph-lacZ,*T_{bkdB}
10	pHB201 P_{acoA} TB2	P_{acoA}, *csn-SP, Ph-lacZ,*T_{bkdB}	40	pMSE3 P_{aprE} TB8	P_{aprE}, *ylqB -SP, Ph-lacZ,*T_{bkdB}
11	pHB201 P_{aprE} TB1	P_{aprE}, *DB, csn-SP, Ph-lacZ,*T_{bkdB}	41	pBE-S P_{acoA} TB7	P_{acoA}, *DB, ylqB -SP, Ph-lacZ,*T_{bkdB}
12	pHB201 P_{aprE} TB2	P_{aprE}, *csn-SP, Ph-lacZ,*T_{bkdB}	42	pBE-S P_{acoA} TB8	P_{acoA}, *ylqB -SP, Ph-lacZ,*T_{bkdB}
13	pMSE3 P_{acoA} TB3	P_{acoA}, *DB, lipA-SP, Ph-lacZ,*T_{bkdB}	43	pBE-S P_{aprE} TB7	P_{aprE}, *DB, ylqB -SP, Ph-lacZ,*T_{bkdB}
14	pMSE3 P_{acoA} TB4	P_{acoA}, *lipA -SP, Ph-lacZ,*T_{bkdB}	44	pBE-S P_{aprE} TB8	P_{aprE}, *ylqB -SP, Ph-lacZ,*T_{bkdB}
15	pMSE3 P_{aprE} TB3	P_{aprE}, *DB, lipA -SP, Ph-lacZ,*T_{bkdB}	45	pHB201 P_{acoA} TB7	P_{acoA}, *DB, ylqB -SP, Ph-lacZ,*T_{bkdB}
16	pMSE3 P_{aprE} TB4	P_{aprE}, *lipA -SP, Ph-lacZ,*T_{bkdB}	46	pHB201 P_{acoA} TB8	P_{acoA}, *ylqB -SP, Ph-lacZ,*T_{bkdB}
17	pBE-S P_{acoA} TB3	P_{acoA}, *DB, lipA -SP, Ph-lacZ,*T_{bkdB}	47	pHB201 P_{aprE} TB7	P_{aprE}, *DB, ylqB -SP, Ph-lacZ,*T_{bkdB}
18	pBE-S P_{acoA} TB4	P_{acoA}, *lipA -SP, Ph-lacZ,*T_{bkdB}	48	pHB201 P_{aprE} TB8	P_{aprE}, *ylqB -SP, Ph-lacZ,*T_{bkdB}
19	pBE-S P_{aprE} TB3	P_{aprE}, *DB, lipA -SP, Ph-lacZ,*T_{bkdB}	49	pMSE3 P_{acoA} TB9	P_{acoA}, *DB, Ph-lacZ,*T_{bkdB}
20	pBE-S P_{aprE} TB4	P_{aprE}, *lipA -SP, Ph-lacZ,*T_{bkdB}	50	pMSE3 P_{acoA} TB10	P_{acoA}, *Ph-lacZ,*T_{bkdB}
21	pHB201 P_{acoA} TB3	P_{acoA}, *DB, lipA -SP, Ph-lacZ,*T_{bkdB}	51	pMSE3 P_{aprE} TB9	P_{aprE}, *DB, Ph-lacZ,*T_{bkdB}
22	pHB201 P_{acoA} TB4	P_{acoA}, *lipA -SP, Ph-lacZ,*T_{bkdB}	52	pMSE3 P_{aprE} TB10	P_{aprE}, *Ph-lacZ,*T_{bkdB}
23	pHB201 P_{aprE} TB3	P_{aprE}, *DB, lipA -SP, Ph-lacZ,*T_{bkdB}	53	pBE-S P_{acoA} TB9	P_{acoA}, *DB, Ph-lacZ,*T_{bkdB}
24	pHB201 P_{aprE} TB4	P_{aprE}, *lipA -SP, Ph-lacZ,*T_{bkdB}	54	pBE-S P_{acoA} TB10	P_{acoA}, *Ph-lacZ,*T_{bkdB}
25	pMSE3 P_{acoA} TB5	P_{acoA}, *DB, yncM-SP, Ph-lacZ,*T_{bkdB}	55	pBE-S P_{aprE} TB9	P_{aprE}, *DB, Ph-lacZ,*T_{bkdB}
26	pMSE3 P_{acoA} TB6	P_{acoA}, *yncM -SP, Ph-lacZ,*T_{bkdB}	56	pBE-S P_{aprE} TB10	P_{aprE}, *Ph-lacZ,*T_{bkdB}
27	pMSE3 P_{aprE} TB5	P_{aprE}, *DB, yncM -SP, Ph-lacZ,*T_{bkdB}	57	pHB201 P_{acoA} TB9	P_{acoA}, *DB, Ph-lacZ,*T_{bkdB}
28	pMSE3 P_{aprE} TB6	P_{aprE}, *yncM -SP, Ph-lacZ,*T_{bkdB}	58	pHB201 P_{acoA} TB10	P_{acoA}, *Ph-lacZ,*T_{bkdB}
29	pBE-S P_{acoA} TB5	P_{acoA}, *DB, yncM -SP, Ph-lacZ,*T_{bkdB}	59	pHB201 P_{aprE} TB9	P_{aprE}, *DB, Ph-lacZ,*T_{bkdB}
30	pBE-S P_{acoA} TB6	P_{acoA}, *yncM -SP, Ph-lacZ,*T_{bkdB}	60	pHB201 P_{aprE} TB10	P_{aprE}, *Ph-lacZ,*T_{bkdB}

Supplemental Table S3: Overview of Sox and IL expression vectors

Nos.	Plasmid nomenclature	Relevant genetic elements	Nos.	Plasmid nomenclature	Relevant genetic elements
1	pMSE3 P_{acoA} S1	P_{acoA}, *DB*, *csn-SP*, Sox, 6xHis,T_{bkdB}	13	pMSE3 P_{acoA} IL1	P_{acoA}, *DB*, *lipA-SP*, IL, 6xHis,T_{bkdB}
2	pMSE3 P_{acoA} S2	P_{acoA}, *csn-SP*, Sox, 6xHis,T_{bkdB}	14	pMSE3 P_{acoA} IL2	P_{acoA}, *lipA-SP*, IL, 6xHis,T_{bkdB}
3	pMSE3 P_{aprE} S3	P_{aprE}, *DB*, *csn-SP*, Sox, 6xHis,T_{bkdB}	15	pMSE3 P_{aprE} IL3	P_{aprE}, *DB*, *lipA-SP*, IL, 6xHis,T_{bkdB}
4	pMSE3 P_{aprE} S4	P_{aprE}, *csn-SP*, Sox, 6xHis,T_{bkdB}	16	pMSE3 P_{aprE} IL4	P_{aprE}, *lipA-SP*, IL, 6xHis,T_{bkdB}
5	pBE-S P_{acoA} S5	P_{acoA}, *DB*, *csn-SP*, Sox, 6xHis,T_{bkdB}	17	pBE-S P_{acoA} IL5	P_{acoA}, *DB*, *lipA-SP*, IL, 6xHis,T_{bkdB}
6	pBE-S P_{acoA} S6	P_{acoA}, *csn-SP*, Sox, 6xHis,T_{bkdB}	18	pBE-S P_{acoA} IL6	P_{acoA}, *lipA-SP*, IL, 6xHis,T_{bkdB}
7	pBE-S P_{aprE} S7	P_{aprE}, *DB*, *csn-SP*, Sox, 6xHis,T_{bkdB}	19	pBE-S P_{aprE} IL7	P_{aprE}, *DB*, *lipA-SP*, IL, 6xHis,T_{bkdB}
8	pBE-S P_{aprE} S8	P_{aprE}, *csn-SP*, Sox, 6xHis,T_{bkdB}	20	pBE-S P_{aprE} IL8	P_{aprE}, *lipA-SP*, IL, 6xHis,T_{bkdB}
9	pHB201 P_{acoA} S9	P_{acoA}, *DB*, *csn-SP*, Sox, 6xHis,T_{bkdB}	21	pHB201 P_{acoA} IL9	P_{acoA}, *DB*, *lipA-SP*, IL, 6xHis,T_{bkdB}
10	pHB201 P_{acoA} S10	P_{acoA}, *csn-SP*, Sox, 6xHis,T_{bkdB}	22	pHB201 P_{acoA} IL10	P_{acoA}, *lipA-SP*, IL, 6xHis,T_{bkdB}
11	pHB201 P_{aprE} S11	P_{aprE}, *DB*, *csn-SP*, Sox, 6xHis,T_{bkdB}	23	pHB201 P_{aprE} IL11	P_{aprE}, *DB*, *lipA-SP*, IL, 6xHis,T_{bkdB}
12	pHB201 P_{aprE} S12	P_{aprE}, *csn-SP*, Sox, 6xHis,T_{bkdB}	24	pHB201 P_{aprE} IL12	P_{aprE}, *lipA-SP*, IL, 6xHis,T_{bkdB}
